# Human Pleural Fluid Elicits Pyruvate and Phenylalanine Metabolism in *Acinetobacter baumannii* to Enhance Cytotoxicity and Immune Evasion

**DOI:** 10.3389/fmicb.2019.01581

**Published:** 2019-07-17

**Authors:** Nyah Rodman, Jasmine Martinez, Sammie Fung, Jun Nakanouchi, Amber L. Myers, Caitlin M. Harris, Emily Dang, Jennifer S. Fernandez, Christine Liu, Anthony M. Mendoza, Veronica Jimenez, Nikolas Nikolaidis, Catherine A. Brennan, Robert A. Bonomo, Rodrigo Sieira, Maria Soledad Ramirez

**Affiliations:** ^1^Center for Applied Biotechnology Studies, Department of Biological Science, College of Natural Sciences and Mathematics, California State University, Fullerton, Fullerton, CA, United States; ^2^Medical Service and Geriatrics Research, Education and Clinical Center (GRECC), Louis Stokes Cleveland Department of Veterans Affairs Medical Center, Cleveland, OH, United States; ^3^Departments of Medicine, Pharmacology, Molecular Biology and Microbiology, Biochemistry, Proteomics and Bioinformatics, Case Western Reserve University School of Medicine, Cleveland, OH, United States; ^4^CWRU-Cleveland VAMC Center for Antimicrobial Resistance and Epidemiology (Case VA CARES), Cleveland, OH, United States; ^5^Fundacioìn Instituto Leloir-IIBBA CONICET, Buenos Aires, Argentina

**Keywords:** *Acinetobacter baumannii*, metabolism, immunoevasion, pleural fluid, pyruvate, phenylalanine, antibiotic resistance

## Abstract

*Acinetobacter baumannii* (*Ab*) is one of the most treacherous pathogens among those causing hospital-acquired pneumonia (HAP). *A. baumannii* possesses an adaptable physiology, seen not only in its antibiotic resistance and virulence phenotypes but also in its metabolic versatility. In this study, we observed that *A. baumannii* undergoes global transcriptional changes in response to human pleural fluid (PF), a key host-derived environmental signal. Differential gene expression analyses combined with experimental approaches revealed changes in *A. baumannii* metabolism, affecting cytotoxicity, persistence, bacterial killing, and chemotaxis. Over 1,220 genes representing 55% of the differentially expressed transcriptomic data corresponded to metabolic processes, including the upregulation of glutamate, short chain fatty acid, and styrene metabolism. We observed an upregulation by 1.83- and 2.61-fold of the pyruvate dehydrogenase complex subunits E3 and E2, respectively. We also found that pyruvate (PYR), in conjunction with PF, triggers an *A. baumannii* pathogenic behavior that adversely impacts human epithelial cell viability. Interestingly, PF also amplified *A. baumannii* cytotoxicity against murine macrophages, suggesting an immune evasion strategy implemented by *A. baumannii*. Moreover, we uncovered opposing metabolic strategies dependent on the degree of pathogenicity of the strains, where less pathogenic strains demonstrated greater utilization of PYR to promote persister formation in the presence of PF. Additionally, our transcriptomic analysis and growth studies of *A. baumannii* suggest the existence of an alternative phenylalanine (PA) catabolic route independent of the phenylacetic acid pathway, which converts PA to phenylpyruvate (PP) and shuttles intermediates into styrene metabolism. This alternative route promoted a neutrophil-evasive state, as PF-induced degradation of PP significantly reduced overall human neutrophil chemotaxis in *ex vivo* chemotactic assays. Taken together, these data highlight *A. baumannii* pathoadaptabililty in response to host signals and provide further insight into the role of bacterial metabolism in virulence traits, antibiotic persistence strategies, and host innate immune evasion.

## Introduction

*Acinetobacter baumannii* is a notorious nosocomial pathogen with mortality rates among patients in intensive care units reported between 21.6 and 67% (Falagas et al., 2006; Peleg et al., 2008; Roca et al., 2012; Park et al., 2013; Inchai et al., 2015; Xiao et al., 2017). *A. baumannii* is responsible for a high number of health-care-associated cases of pneumonia, bacteremia, and urinary, skin, or surgical-derived infections (Peleg et al., 2008; Roca et al., 2012; Wong et al., 2017). This Gram-negative bacterium has also garnered considerable clinical significance as a result of its intrinsic antibiotic resistance mechanisms, innate ability to persist for long periods upon desiccation and starvation, and capability to acquire foreign resistance determinants from the surrounding environment (Jawad et al., 1998; Peleg et al., 2008; Adams et al., 2010; Sahl et al., 2013; Chapartegui-Gonzalez et al., 2018). Because of its growing antibiotic resistance profile, the World Health Organization (WHO) has assigned *A. baumannii* a threat level “critical” on the Priority Pathogens List for new antimicrobial research and development (WHO, 2017).

Apart from its dynamic antibiotic resistance, *A. baumannii* has also been shown to be extremely persistent under many conditions (Jawad et al., 1998; Roca et al., 2012; Mortensen and Skaar, 2013; Juttukonda et al., 2016). Unique among Gram-negative pathogens, *A. baumannii* persists for a prolonged period of time in the environment and during patient colonization in spite of antimicrobial exposure, nutrient deprivation, or the presence of immune effectors. Modifications in metabolism and nutritional needs have been linked to the ability of *A. baumannii* to persist and adapt to a hostile host environment (Mortensen and Skaar, 2013). In fact, *A. baumannii* metabolic flexibility was shown to overcome host nutrient limitation *via* urea carboxylase-induced calprotectin resistance; differential expression of *zur* and *fur* transcriptional regulators; upregulation of Mn^2+^, Zn^2+^, Fe^3+^ uptake systems; and increased secretion of iron-scavenging siderophores (Juttukonda et al., 2016). Additionally, cytolytic activity and immunoevasive mechanisms of *A. baumannii* have also been linked to metal acquisition and phenylacetic acid (PAA) metabolism (Bhuiyan et al., 2016; Fiester et al., 2016).

Pathogens maintain a balance between virulence and persistence during host infection to succeed while competing for nutritional factors and host immune effectors and secreting advantageous toxins and proteins. Human serum albumin (HSA), the main blood protein component and an important marker of host nutritional and inflammatory status, triggers the differential expression of a number of key genes involved in the survival and persistence of *A. baumannii* (Don and Kaysen, 2004; Quinn et al., 2018b). Pleural fluid (PF), an HSA-containing medium that primarily functions to lubricate pleurae during respiratory movements, is continuously produced by intercostal arteries and removed by the lymphatic system. Disruption of this flow can result in an abnormal amount of fluid in the chest cavity known as a pleural effusion, a frequent sign of underlying disease to which bacterial pneumonia constitutes the second leading cause in the United States with an annual incidence of 300,000 (Light, 2006). Hospitalized patients diagnosed with bacterial pneumonia, one of the most common clinical manifestations of *A. baumannii*, in 40% of cases develop pleural effusion (Chen et al., 2001; Costa et al., 2001; Joly-Guillou, 2005; Light, 2006). Accordingly, PF constitutes a host component that this nosocomial pathogen is in many instances obliged to cope with. Both the previously reported signaling properties of HSA and the presence of immune cells and effectors in PF contents lead us to hypothesize that PF provides a diverse set of environmental cues for bacterial adaptation to harsh conditions within the host. Therefore, we investigated the notion that PF could trigger adaptive responses in the pathophysiology of *A. baumannii* during respiratory infection. In this study, we aimed to assess changes in the metabolic profile of *A. baumannii* upon exposure to PF and their possible role in the establishment of survival strategies. To test our hypothesis, three model strains (A118, A42, and AB5075) with varying degrees of antibiotic susceptibility and virulence were used (Ramirez et al., 2010; Ramirez et al., 2011; Vilacoba et al., 2013; Jacobs et al., 2014; Traglia et al., 2016). A118, the least antibiotic-resistant and virulent strain, was selected to perform transcriptomic analysis, metabolic microarrays, and virulence testing as the strain has been previously used as a model system (Ramirez et al., 2010; Ramirez et al., 2012; Traglia et al., 2016; Domingues et al., 2018; Martinez et al., 2018; Quinn et al., 2018a,b,c; Domingues et al., 2019). A42, the mildly resistant and virulent strain, and AB5075, the most resistant and virulent, were then used to supplement our assessment of the *A. baumannii* metabolic profile in response to PF. Our findings revealed unique insights into the complexity of metabolic adaptive responses in this pathogen.

## Materials and Methods

### Bacterial Strains, Mammalian Cell Lines, and Human Fluid

*Acinetobacter baumannii* A118, A42, and AB5075 strains were used (Ramirez et al., 2011; Jacobs et al., 2014; Quinn et al., 2018a,c) as bacterial models (Ramirez et al., 2010; Jacobs et al., 2012; Ramirez et al., 2012; Jacobs et al., 2014; Traglia et al., 2016; Quinn et al., 2018a,c; Table 1). *Escherichia coli*-TET-R and *Staphylococcus aureus* USA300 were also used in pathogenicity-determining experiments (Knauf and Nester, 1982; Diekema et al., 2014). Bacterial cells were plated from -80°C storage on cystine lactose electrolyte deficient (CLED) agar and later cultured in Luria–Bertani (LB) broth at 37°C and/or the different tested conditions.

**Table 1:** Characteristics of the strains used in the study.

Strain	Site of isolation	Virulence	Resistance	References
A118	Blood	Low virulence	Highly susceptible	Ramirez et al., 2010
A42	Endotracheal aspirate	Mildly virulent	Multidrug resistant	Vilacoba et al., 2013
AB5075	Tibia/Osteomyelitis	Extremely virulent	Extremely drug resistant	Jacobs et al., 2014

American Type Culture Collection (ATCC) HeLa cells and RAW 264.7 macrophages were used for cytotoxicity assay. Cell lines were routinely grown in Dubelcco’s modified Eagle’s minimum essential medium (DMEM) (Invitrogen), supplemented with 10% (v/v) fetal calf serum (ATCC, Manassas, VA, United States) as well as penicillin/streptomycin and incubated at 37°C in 5% CO_2_ humidified atmosphere. Human neutrophils were obtained from a certified vendor (Astarte Biologicals Inc., Bothell, WA, United States) and used for the neutrophil chemotaxis assay.

The human PF sample used in the present study was acquired from Innovative Research, MI, which is a certified vendor that obtains human samples from Food and Drug Administration (FDA)-approved facilities. LB supplemented with 4% PF was used for all PF conditions; the concentration was previously established in Martinez et al. (2018). The ability of A118 to grow in 100% PF was assessed, and it was observed that A118 can grow in 100% of this human fluid (data not shown).

All procedures performed in this study were in accordance with the CSUF Institutional Biosafety Committee Approval plan (DBH117-01) and are in compliance with the National Institutes of Health (NIH), Centers for Disease Control and Prevention (CDC), Occupational Safety and Health Administration (OSHA0), and other environmental and occupational regulations.

### RNA Extraction, Sequencing, and Transcriptomic Analysis

In 2010, the strain A118 was established as a study model, and it has recently been used for transcriptomic analysis for a human component; this led us to select it for transcriptomic analysis in the presence of PF (Ramirez et al., 2010; Quinn et al., 2018b). A118 cells were cultured in LB broth with or without PF and incubated with agitation for 18 h at 37°C. Overnight cultures were then diluted 1:10 in fresh LB broth and incubated with agitation for 7 h at 37°C. RNA was immediately extracted following the TRI REAGENT^®^Kit (Molecular Research Center, Inc., Cincinnati, OH, United States) as previously described (Quinn et al., 2018b).

RNA sequencing was outsourced to Novogene (Novogene Corporation, CA, United States) for mRNA-seq analysis, which includes rRNA depletion, library preparation through Next^®^Ultra^TM^ RNA Library Prep Kit for Illumina (New England Biolabs), and HiSeq 2500 paired-end 150 base pair (bp) sequencing. Trimming of low-quality bases at the ends of the reads to a minimum length of 100 bp and removal of Illumina adaptor sequences were performed using Trimmomatic (version 0.32). Quality of the reads before and after trimming was assessed using FastQC^1^. Reads were then aligned to the contigs of the genome of *A. baumannii* A118 strain using the Burrows–Wheeler Alignment (BWA) software (Li and Durbin, 2010), yielding >91% unambiguously mapped reads per library. Alignments were visualized using the Integrated Genome Viewer software (Thorvaldsdottir et al., 2013). Read counts per gene were calculated using FeatureCounts (Quinlan and Hall, 2010), and differential expression analysis was performed using DEseq (Anders and Huber, 2010). Both principal component analysis (PCA) and gene expression heat map with clustering dendrograms of the RNA-seq data analysis showed that the biological replicates of LB and PF treatments were effectively clustered into two well-defined groups (Supplementary Figure S1). Transcriptomic data of metabolic-related genes were considered for the present study. Features exhibiting false discovery rate (FDR) <0.05 were considered statistically significant.

RNA-seq data generated as a result of this work have been deposited to Gene Expression Omnibus (GEO) with the accession number GSE131949.

### Phenotype Microarray Assay and Analysis

Bacterial strains were cultured in LB and LB + 4% PF at 37°C for 18 h. Inoculation and preparation were performed *via* manufacturer instructions (Biolog, Hayward, CA, United States) with exception of using isolated cells from overnight culture to inoculate sole carbon sources. PM1, PM2A (both carbon sources), and PM3 (nitrogen source) were used. Plates were incubated under aerobic conditions for 24 h at 37°C, and results were read at OD_590_ using the multimodal plate reader SpectraMax3.

### Cell-Free Culture Media Preparation

Cell-free culture media (CFCM) was obtained as previously described by allowing *A. baumannii* strains to grow for 48 h at 37°C under shaking conditions (200 rpm) in LB, PF, LB + 1% pyruvate (PYR), PF + 1% PYR, LB + 0.02% phenylalanine (PA), PF + 0.02% PA, LB + 0.02% PAA, PF + 0.02% PAA, LB + 0.02% phenylpyruvate (PP), PF + 0.02% PP, and culture (Ramsey et al., 2016; Castellanos et al., 2019). At the end of the incubation period, bacterial cultures were centrifuged for 10 min at 7,000 × *g*. The supernatant was filtered through a Steriflip Vacuum Filtration System with Millipore Express PLUS Membrane (0.22 μm) (Millipore, #SCGP00525).

PYR (1%) was used, since it was previously shown to induce a significant increase in the exoproteome profile of *S. aureus*, enhancing its pathogenicity (Harper et al., 2018); 0.02% PA, PAA, and PP CFCM were used due to the minimal growth seen in A118 growth curves of PAA with or without PF in both minimal media growth curves and in LB. To be consistent, 0.02% was used in CFCM studies for all three conditions.

### Cytotoxicity Assay

The CFCM of A118 LB, PF, LB + 1% PYR, and PF + 1% PYR were collected and diluted with colorless DMEM to 0.2–50% CFCM. ATCC HeLa cells and RAW 264.7 macrophages were diluted to a final concentration of 1 × 10^6^ cells/ml in colorless DMEM. HeLa and RAW suspensions were then added in a final volume of 50 μl/well to Nunclon^TM^ Delta Surface opaque 96-well microplate (ThermoScientific). HeLa and RAW cells were then intoxicated for 1 h at 37°C, 5% CO_2_, with 50 μl of experimental CFCM conditions. CellTiter-Glo^®^Reagent was prepared according to the manufacturer’s instructions (Promega, Madison, WI, United States), and adenosine triphosphate (ATP) (Sigma) was resuspended in colorless DMEM to generate a standard curve. CellTiter-Glo^®^Reagent (100 μl) was added to each experimental and standard curve well and then placed on an orbital shaker for 2 min. Following mixing, plates were incubated at room temperature for 10 min to stabilize the luminescent signal. The viability of RAW and HeLa cells was measured at room temperature using the “all” luminescence function of SpectraMax M3.

### Dehydrogenase Activity Assays of Cell Soluble Extracts

Dehydrogenase activity was measure in cell soluble extracts of A118 strains with and without PF. Aliquots (1 ml) were pelleted and resuspended in 80 μl of resuspension buffer (100 mM 4-(2-hydroxyethyl)-1-piperazineethanesulfonic acid (HEPES), 1 M MgCl_2_, pH 7.5). Soluble extracts treated with 0.8 mg lysozyme were added, and samples were incubated at 37°C for 30 min. Samples were centrifuged at 12,000 rpm for 10 min at 4°C. For malate dehydrogenase (MDH) activity, 16 μl of supernatant was added to 184 μl of forward reaction buffer (100 mM HEPES pH 7.5, 10 mM NAD^+^, 100 mM L-malic acid, 1 mM DTT, 10 mM MgCl_2_) per well. To test 2-oxoglutarate dehydrogenase, α-ketoglutaric acid (100 mM) was added instead of L-malic acid. All dehydrogenase assays were conducted in triplicate. The absorbance was analyzed using spectrophotometric analysis (SpectraMax M3) at 30°C with an excitation of 340 nm and an emission of 460 nm. Measurements were taken at time 0 and 10 min later. During that 10 min, the plate was stored in an incubator at 37°C.

### Persistence Assay

The persistence assay was performed as described previously (Muller et al., 2017) with slight modifications. Briefly, A118, A42, and AB5075 overnight cultures (stationary-phase cultures) grown in LB, PF, LB + 1% PYR, and PF + 1% PYR were treated with ofloxacin (OFX) at a final concentration of 3X MIC (μg/ml) for 5 h at 37°C in 96 multiwell microplates. A control treatment with sterile water was performed in parallel. The number of colony-forming units (CFUs) was determined by plate counts in each case. The persister fraction is defined as the number of surviving cells after treatment with OFX divided by the number of cells after control treatment (water). The experiments were performed in triplicate. Statistical analysis (Mann–Whitney test) was performed using GraphPad Prism (GraphPad software, San Diego, CA, United States). A *P*-value < 0.05 was considered significant.

### Bacterial Killing Assay

Killing assays were carried out as previously described with minor changes (Weber et al., 2015; Quinn et al., 2018b). Briefly, A118 and *E. coli*-TET as predator and prey, respectively, were used. A118 LB, PF, LB + 1% PYR, PF + 1% PYR, and *E. coli* cultures were grown and normalized. A118 in the different conditions and *E. coli* were mixed at a predator–prey 1:1 ratio, and 5 μl was spotted on a dry LB agar plate. After 4 h, the spot was resuspended in 1 ml of PBS and 10-μl serial dilutions were plated on LB agar plate with tetracycline (30 μg/ml). Growth of the prey was recorded after incubation at 37°C for 24 h.

### Aromatic Sugar and Carboxylic Acid Utilization Assays

Cultures were grown in LB broth induced with or without PF overnight in 37°C with shaking. Growth assays were conducted on 96-well plates in triplicate in Minimal Medium M9 supplemented with 1 μM MgSO_4_, with 0.2% PA, 0.2% PAA, 0.2% PP, 0.2% fumarate, 0.2% malate, and 0.2% succinate. Additional nutrient assays were conducted in LB supplemented with 1% PYR, 0.2% PAA, 0.2% PA, 0.2% PP, 0.02% PA, 0.02% PAA, and 0.02% PP. Wells containing 294 μl of media and 6 μl of culture were incubated for 18 h in 37°C with medium shaking. Growth was measured at OD_600_ every 20 min. Mann–Whitney test was performed for statistical analysis.

### Quantitative Reverse Transcription Polymerase Chain Reaction (qPCR)

Previously extracted and DNase-treated RNA from *A. baumannii* strain A118 grown in LB or 4% PF were synthesized to cDNA using the manufacturer protocol provided within the iScript^TM^ Reverse Transcription Supermix for qPCR (Bio-Rad, Hercules, CA, United States). The cDNA concentrations were measured with a DeNovix DS-11+ spectrophotometer; each sample was then diluted to a concentration of 50 ng/μl. qPCR was conducted using the iQ^TM^ SYBR^®^Green Supermix through the manufacturer’s instructions. At least three biological replicates of cDNA were used and were run in quadruplet. All samples were then run on the CFX96 Touch^TM^ Real-Time PCR Detection System (Bio-Rad, Hercules, CA, United States).

The transcript levels of each sample were normalized to the *recA* rRNA transcript levels for each cDNA sample. The relative quantification of gene expression was performed using the comparative threshold method 2^-ΔΔCt^. The ratios obtained after normalization were expressed as folds of change compared with cDNA samples isolated from bacteria cultures on LB. Statistical analysis (Mann–Whitney test) was performed using GraphPad Prism (GraphPad software, San Diego, CA, United States). A *P*-value < 0.05 was considered significant.

### Neutrophil Chemotaxis Assay

The neutrophil chemotaxis assay was performed as described previously with slight modifications (Henkels et al., 2011; Kaur and Singh, 2013; Bhuiyan et al., 2016). Herein, 25 ml of Roswell Park Memorial Institute Medium (RMPI; Thermo Fisher 22400), 2.5 ml fetal bovine serum (FBS; Thermo Fisher A3160601), 500 μl of 100 U/ml penicillin–streptomycin, and 22 ml of Hanks’ balanced salt solution (HBSS; Thermo Fisher 14025) were combined to make the chemotaxis buffer. Twenty-four-well Olympus polycarbonate tissue culture plates with 8-μm pore size membranes were used (Genesee Cat #25-289). The chemotaxis buffer (200 μl) was combined with 200 μl of CFCM of each condition. Eight different A118 CFCM conditions were tested: LB only, PF + LB, 0.02% PA in LB, 0.02% PAA in LB, 0.02% PP in LB, 0.02% PA in PF + LB, 0.02% PAA in PF + LB, and 0.02% PP in PF + LB. A control treatment of each of the medium conditions without CFCM was performed in parallel. Neutrophils (200 μl; 10^5^ cells/ml) were added to the semipermeable well inserts. The plate was incubated for 1 h in 5% atmospheric CO_2_ at 37°C. Cells were counted from 20-μl representative samples of each well. Chemotaxis index was calculated by dividing the number of neutrophils in the test well by the number of neutrophils in the control well. The experiments were performed in triplicates.

### Statistical Analysis

Data are expressed as mean ± standard deviation (SD). Statistical analyses [Mann–Whitney test, multiple *t*-test, one-way analysis of variance (ANOVA), and two-way ANOVA] were performed using GraphPad Prism (GraphPad software, San Diego, CA, United States). A *P*-value < 0.05 was considered significant. All reported results are statistically significant unless otherwise stated.

## Results

### PF Shifts Metabolic Gene Expression of *A. baumannii* Strain A118

Transcriptome analysis of *A. baumannii* strain A118 in the presence of human PF revealed a comprehensive differential profile of 2,232 genes with an FDR-adjusted *P*-value < 0.05 (Supplementary Figure S2). Consistent with the composition complexity of PF, this transcriptional response was notably more pronounced than that of the 111 statistically significant differentially expressed genes (DEGs) previously observed in strain A118 exposed to HSA (Quinn et al., 2018b). Genes associated with metabolic processes represented more than 55% of our DEGs (*n* = 2,232). Genes involved in glutamate, short chain fatty acid, and styrene metabolism, as well as the PYR dehydrogenase complex were upregulated.

### PF Promotes Conversion of PYR to Specific Metabolic Precursors

**FIGURE 1:**
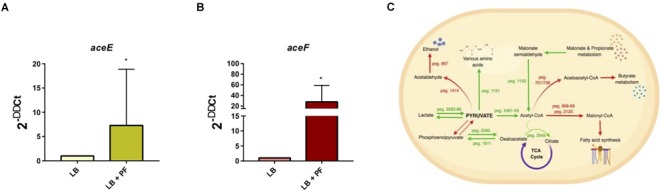
PF augments transcriptomic response of PYR metabolism-associated genes. **(A)** qPCR of A118 strain genes associated with the PDH complex, *aceE*, and **(B)** *aceF* in the presence of PF. Fold changes were calculated using double ∆Ct analysis. At least three independent samples were used, and four technical replicates were performed from each sample. Statistical significance (^∗^*P* < 0.05) was determined by the Mann–Whitney *t*-test (*n* = 3 to 4). **(C)** DEGs associated with PYR metabolism under exposure to PF. Red words and arrows indicate downregulated, statistically significant genes (adjusted *P* < 0.05). Green words and arrows indicate upregulated, statistically significant genes (adjusted *P* < 0.05). Dotted arrows indicate DEGs that were not statistically significant. Black arrows indicate genes that were not differentially expressed under PF treatment. Dashed lines indicate a second substrate utilized in the enzymatic conversion to next metabolite of the pathway.

We observed an interesting effect in the expression of genes associated to PYR metabolism in our transcriptomic data. We found that under PF exposure, the expression of genes peg.2461–63 encoding three components of the pyruvate dehydrogenase (PDH) complex were upregulated (Supplementary Table S1). The PDH complex of *A. baumannii*, which is responsible for acetyl-CoA production, is composed of three subunits: PDH (E1), dihydrolipoamide acetyltransferase subunit (E2), and the dihydrolipoamide dehydrogenase subunit (E3). The gene encoding the E1 subunit of the PDH complex, *aceE*, was upregulated by a fold change of 2.03, and the E2 subunit, *aceF*, was upregulated by a fold change of 2.61 (Supplementary Table S1). qPCR confirmed these results (Figure 1A,B), thus supporting that conversion of PYR into acetyl-CoA production is indeed favored in the presence of PF (Figure 1C).

**FIGURE 2:**
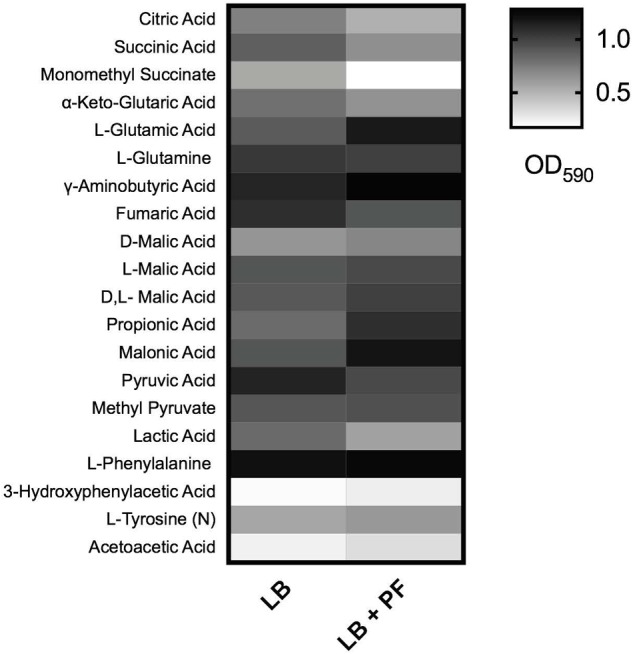
Phenotype microarray. Heat map of phenotype microarray demonstrating growth of *Acinetobacter baumannii* A118 strain, with and without PF on selected carbon sources. L-tyrosine acted as a nitrogen source, denoted by (N) (*n* = 1).

Given that acetyl-CoA is a significant metabolite for fatty acid synthesis, these latter PF-mediated transcriptional effects could, in principle, increase *A. baumannii* lipid metabolism. However, we observed that the expressions of many of the enzymes involved in the fatty acid synthetic pathway were downregulated in response to PF. Four genes associated with exogenous fatty acid incorporation during phospholipid membrane synthesis in *E. coli*, including *fadD, plsB*, and *plsC* (Green et al., 1981; Coleman, 1992; Yao and Rock, 2013), were upregulated in response to PF (Supplementary Table S1), suggesting a shift away from *de novo* phospholipid synthesis to conserve energy. Our transcriptomic data also revealed a possible shift toward short chain fatty acid degradation pathways, as eight genes associated with the propionate degradation route of fatty acid metabolism and four genes involved in malonate utilization were upregulated in the presence of PF (Supplementary Table S1). Phenotype microarray data further supported this in part, revealing a 1.32-fold and 1.25-fold increase in growth of A118 in malonate and propionate, respectively, in the presence of PF (Figure 2). Because propionate and malonate can serve as a single carbon source through *prp* operon and *acnB/D*-mediated conversion into PYR and succinate, where the latter can be shuttled into the TCA to amplify energy production (Suvorova et al., 2012), we hypothesized that the PF-induced shift to short chain fatty acid degradation may contribute to the ability of *A. baumannii* to persist within the host.

Other systems differentially expressed under PF induction involved in PYR synthesis and utilization (Figure 1C) include the phosphoenolpyruvate carbohydrate phosphotransferase system and transamination. Ten aminotransferase genes were upregulated, including the 1.58-fold upregulation of the ϕ-amino acid PYR aminotransferase gene, peg.1151, which uses PYR to transaminate a variety of α-amino acids, monoamines, and diamines (Yonaha et al., 1983). *gabT*, which was upregulated by 8.19-fold in the presence of PF, can also use PYR in its transamination activity to produce succinate semi-aldehyde and L-alanine (Supplementary Table S1).

Anaerobic utilization and synthesis of PYR are associated with lactate metabolism. The lactate metabolism locus, which consists of the L-lactate permease, two lactate dehydrogenases, and a lactate response regulator (peg.3583–3586), was upregulated by PF (Supplementary Table S1). L-lactate permease is responsible for transporting lactate across the membrane, and lactate dehydrogenase oxidatively converts lactate into PYR (Figure 1C). In patients with severe pneumonia, anaerobic metabolism and subsequent lactic acid levels are elevated (Liu et al., 2015). This shift in metabolism increases the availability of lactic acid, which may be used by *A. baumannii* as a carbon source to produce PYR under PF induction. However, growth curves did not demonstrate a significant increase in bacterial growth in LB supplemented with PYR (Supplementary Figure S3A) in the presence of PF. The enhanced gene expression of PDH, lactate dehydrogenase, propionate metabolism, and PYR aminotransferases suggest that in the presence of PF, *A. baumannii* A118 displays a yet-unknown strategy involving PYR.

### PF and PYR Affect *A. baumannii* Cytotoxicity Against Human Epithelial Cells

The global differential expression of genes associated with metabolism elicited by PF suggested a “survival strategy,” where PYR’s role in energy production is secondary to an inducive role of fortification and virulence. PYR has been reported as an inducer of pathogenicity in *S. aureus* (Harper et al., 2018); however, no study, to our knowledge, has reported this phenomenon in Gram-negative pathogens linked with a differential metabolic profile in response to a serous fluid. We hypothesize that the metabolite triggers an adaptive response that strengthens the ability of *A. baumannii* to cope with components of PF that threaten survival (i.e., phagocytic, granulocytic, and neutralization agents). To assess whether such an adaptive response involves secretion of protein compounds or molecules that enhance pathogenesis by impacting hosts components, CFCM of *A. baumannii* A118 grown in LB with or without the addition of PF, either in the presence or in the absence of PYR, was obtained and used in cytotoxicity assays.

**FIGURE 3:**
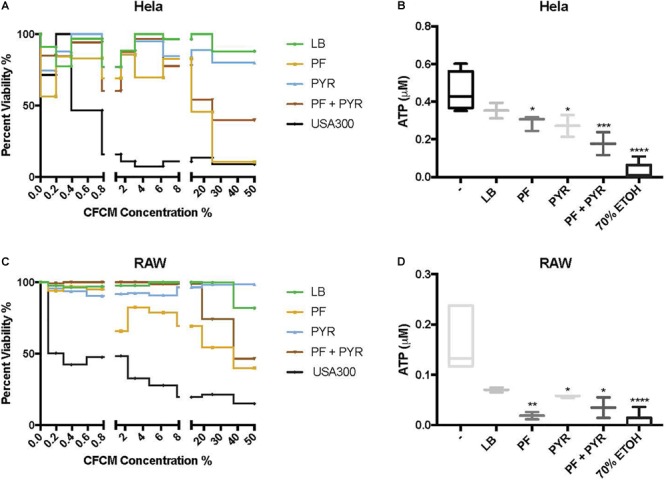
PF and PYR enhance cytotoxicity of A118 against human epithelial cells and murine macrophages. **(A)** Percent viability of HeLa epithelial cells and **(C)** RAW macrophages after 1-h intoxication with concentrations of A118 LB, PF, PYR, or PF + PYR CFCM from 0 to 50%. Viability proportions were determined as the concentration of ATP in experimental treatments divided by the ATP concentration of untreated mammalian cells. *Staphylococcus aureus* USA300 was used as a control to determine efficacy of experiment. **(B)** Viability, defined by ATP μM, of HeLa epithelial cells and **(D)** RAW macrophages upon treatment with 1.56% LB broth, LB CFCM, PF CFCM, PYR CFCM, and PF + PYR CFCM. 70% ethanol (ETOH) was used as cell death control. LB broth was used for determining statistical significance using one-way ANOVA analysis by Sidak’s comparison (*n* = 3–12; ^∗^*P* < 0.05; ^∗∗^*P* < 0.01; ^∗∗∗^*P* < 0.005; ^∗∗∗∗^*P* < 0.0001).

Human epithelial cells (HeLa) were exposed to increasing concentrations of CFCM isolated from *A. baumannii* A118 grown with or without PF, in the absence or presence of 1% PYR as used before (Harper et al., 2018). For both PF and PF + PYR conditions, we observed that as the CFCM concentration increased, HeLa percent viability decreased (Figure 3A). To assess the significance of the observed effect, we compared the extent of the *A. baumannii* A118 cytotoxicity with that of a control performed with CFCM of *S. aureus* USA300, a bacterium known to secrete several hemolytic and cytolytic peptides and leukocidins that contribute to its potent virulence (Kobayashi et al., 2011). Interestingly, we observed that while LB CFCM from *A. baumannii* had minimal cytotoxicity, PF CFCM began to approach *S. aureus* USA300 cytotoxicity levels at 12.5% CFCM, thus demonstrating a PF-mediated enhancement of *A. baumannii* A118 cytotoxicity. On the other hand, PYR alone did not produce a dose-dependent cytotoxic effect against HeLa cells. However, the combination of PF + PYR indeed reduced HeLa viability below 50% once reaching a concentration of 25% CFCM.

We observed the cytotoxic effect induced by *S. aureus* USA300 to plateau well below 50% viability after HeLa exposure to 1.56% CFCM (Figure 3A). Therefore, this percentage (1.56% CFCM) was used to determine relative pathogenicity of *A. baumannii* under various study conditions. Viability of HeLa cells was determined by the amount of ATP present. Interestingly, for both the PF and PYR CFCM conditions, a minor 1.22-fold reduction in ATP was observed, relative to LB CFCM (Figure 3B). When A118 was induced by both PF and PYR, a significant 2.55-fold reduction (*P* < 0.01) in viability occurred, suggesting that, at non-saturating concentrations of CFCM (1.56%), both PF and PYR effects simultaneously work to enhance *A. baumannii* cytotoxicity against human epithelial cells (Figure 3B).

To further assess the role of PF and PYR in *A. baumannii* pathogenesis, we exposed RAW 264.7 macrophages to A118 LB CFCM, PF CFCM, PYR CFCM, and PF + PYR CFCM. Macrophages are the most abundant white blood cell type in PF and are the first responding immune effectors to *A. baumannii* (Garcia-Patino et al., 2017). Consistently with the interaction observed against HeLa, as CFCM concentrations increased, the cytotoxic effect of PF and PF + PYR against RAW macrophages also increased (Figure 3C). PYR CFCM alone did not produce this effect.

Analysis of 1.56% CFCM conditions revealed a 2.4-fold decrease in ATP of LB CFCM-exposed RAW cells (Figure 3D), indicating that A118 constitutively secretes cytotoxic peptides against murine macrophages. However, when A118 was induced by PF, its CFCM triggered a drastic 9.06-fold reduction (*P* < 0.01) in RAW cell viability, almost surpassing the 70% ETOH cell death condition in mean ATP luminescence. We observed only a slight reduction in ATP upon exposure to PYR CFCM compared to LB CFCM. However, PF + PYR CFCM resulted in a stronger decrease in RAW viability. This suggests that the cytotoxic effects of peptides and molecules secreted by A118 against RAW macrophages are amplified by PF, but not by PYR.

### Upon Exposure to PF, PYR Rescues Persistence in *A. baumannii*-Susceptible Strain A118

Persistent bacterial cells, which are a small subpopulation within a susceptible population, can resist antibiotic action, causing treatment failure and relapse of a bacterial infection without having acquired a genetic resistance trait. Given the amplification of cytotoxicity with PYR and PF against human epithelial cells and the enhanced cytotoxicity observed against murine macrophages with PF, we evaluated the occurrence of persister cells upon exposure to a high concentration of antibiotics.

**FIGURE 4:**
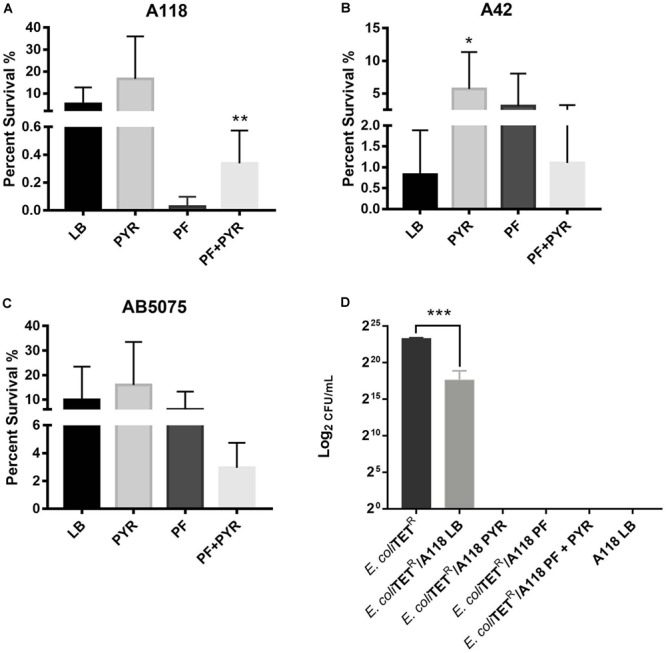
Pyruvate’s role in persister cell formation, and PYR and PF increase bacterial killing activity of strain A118. **(A)** Percentage of surviving cells in LB, PF, LB + 1% PYR, and PF + 1% PYR of strain A118, **(B)** strain A42, and **(C)** strain AB5075. Persister cells are defined as the number of surviving cells after treatment with OFX divided by the number of surviving cells after treatment with water. Statistical significance (^∗^*P* < 0.05; ^∗∗^*P* < 0.005) was determined using unpaired *t*-test (*n* = 3), comparing LB vs. PYR and PF vs. PF + PYR. **(D)** Bacterial killing assay using *Escherichia coli* TOP10-TET (prey) and A118 (predator) grown under the different treatments (LB, PF, LB + 1% PYR, and PF + 1% PYR). Differences in survival of bacterial (*E. coli*-TET prey) colonies are shown in log_2_CFU/ml. Statistical significance was determined using one-way ANOVA analysis (^∗^*P* < 0.05; ^∗∗^*P* < 0.01; ^∗∗∗^*P* < 0.005; ^∗∗∗∗^*P* < 0.0001), and experiments were performed in triplicates.

Persistence assays using high concentrations of OFX (3X MIC) of A118 in LB, PF, LB + PYR, and PF + PYR were performed. We observed that exposure to PF reduced production of persister cells by 24.21-fold (Figure 4A). However, compared to PF alone, PF supplementation with PYR increased the occurrence of persister cells, indicating a rescuing role for PYR in presence of PF (Figure 4A). These data suggest that PYR is used by A118 to generate substrates that can modulate the formation of persister cells, allowing it to survive in nutritionally depriving environments such as PF.

To further test the effect of PYR and/or PF on persistence, A42 and AB5075 strains were also tested. In A42, supplementation of LB with PYR significantly amplified persistence by 6.90-fold, a trend similar to A118. However, unlike A118, PF seemed to induce persistence in A42 (Figure 4B). We also observed a decrease in persistence when PF was supplemented with PYR, indicating an absence of the synergy observed in A118. Given the intrinsic resistance possessed by A42, this suggests that the amplifying persistence relationship of PF + PYR is a survival mechanism used by more susceptible, less virulent strains of *A. baumannii*.

In AB5075, we also observed a decrease in persistence when PF was supplemented with PYR (Figure 4C), demonstrating the same lack of synergy observed in A42. AB5075 is the most resistant and virulent of the three strains used in our model, and, compared to PF alone, the lack of persistence amplification of PF supplemented with PYR suggests that the synergy mechanism between the two substances has an effect only in less pathogenic strains.

To study the effect of PF and PYR on *A. baumannii* virulence, bacterial killing assays were performed against *E. coli*-TET-resistant prey. A118 under the different treatments (LB, PF, LB + PYR, and PF + PYR) was used as the predator to test enhanced virulence and persistence by PF-PYR synergy in clinically susceptible strains of *A. baumannii*. We observed that A118 LB + PYR exhibited increase killing activity, by 400,000-fold, compared with LB alone (Figure 4D), and we also observed the same level of killing activity of A118 grown in PF with or without PYR supplementation (Figure 4D). Our transcriptomic data further showed that three of the genes encoding the type VI secretion system (T6SS), *tssH, vgrG_1*, and *vgrG_5*, were upregulated. Recent studies have revealed that TssH participates in translocation of TssB and TssC, two components of the T6SS contractile tube sheath, into the periplasm. Assembly, contraction, and subsequent release of releasing its effector proteins (Hcp and VgrG) are essential for the T6SS final purpose—specifically to kill bacterial competitors in close proximity (Basler et al., 2012; Russell et al., 2014). The strengthened ability of A118 to kill *E. coli* elicited by both PF and PYR demonstrated that the combination of these two stimuli contributes to the competitive advantage of *A. baumannii*. These results are also in line with modulation of other *A. baumannii* fitness traits such as the enhanced cytotoxicity (Figure 3A–D) and the amplified persistence observed in A118 with PF and/or PYR (Figure 4A).

### An Alternate PA Catabolic Pathway Is Favored Under PF Induction of *A. baumannii*

The PAA catabolic pathway is well studied in *A. baumannii* and contributes to host immune effector evasion and virulence (Teufel et al., 2010; Bhuiyan et al., 2016; Kroger et al., 2016). The *paa* locus is regulated by global virulence regulators, such as GacS and H-NS (Teufel et al., 2010; Bhuiyan et al., 2016; Kroger et al., 2016). Our transcriptomic data revealed that all but three genes of the *paa* locus were downregulated under PF induction (Supplementary Table S1). The genes encoding monoamine oxidase (peg.705) and phenylacetylaldehdye dehydrogenase (*feaB*) were downregulated, suggesting that PA oxidation to PAA and its subsequent catabolism are not favored in the presence of PF. This was further substantiated by the downregulation of the genes encoding succinyl-CoA synthetase and succinyl-CoA ligase (Supplementary Table S1), which are responsible for TCA cycle utilization of succinyl-CoA, a final product of the PAA catabolic pathway.

**FIGURE 5:**
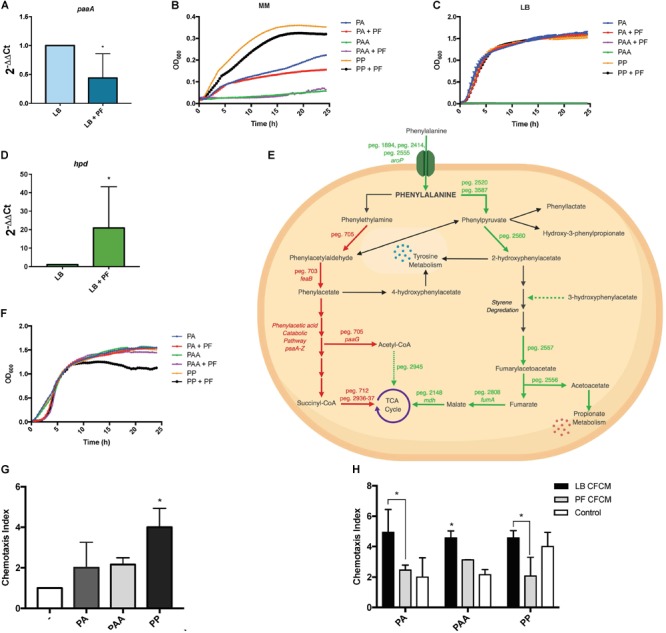
PA alternative utilization changes due to PF induction and its impact on human neutrophil chemoattraction. **(A)** qPCR of A118 *paaA* in the presence of PF. **(B)** Minimal media growth curves of strain A118 in 0.2% PA, 0.2% PAA, or 0.2% PP with or without PF. **(C)** Growth curves of strain A118 in LB supplemented with 0.2% PA, 0.2% PAA, or 0.2% PP with or without PF (*n* = 3). **(D)** qPCR of A118 *hpd*. For both real-time qPCRs, fold changes were calculated using double ∆Ct analysis, and at least three independent samples were used with four technical replicates performed from each sample. Statistical significance (^∗^*P* < 0.05) was determined by Mann–Whitney *t*-test (*n* = 3 to 4). **(E)** Genes differentially expressed by PF connected to PA catabolism. Red words and arrows depict statistically significant downregulated genes (adjusted *P* < 0.05). Green words and arrows depict statistically significant upregulated genes (adjusted *P* < 0.05). Dotted arrows indicate DEGs that were not statistically significant. Black arrows indicate genes that were not differentially expressed under PF treatment. Dashed arrows following a metabolite indicate substrates that showed increased utilization in phenotype microarrays by A118 and lack a correlating differential gene expression in transcriptomic data. **(F)** Growth curves of strain A118 in LB supplemented with 0.02% PA, 0.02% PAA, or 0.02% PP with or without PF. 0.02% was selected as the concentration for PA and its derivatives for testing in order to mimic the mean concentration of amino acid nitrogen in normal human blood (Schaeffer et al., 1975; Lamonica et al., 2012). Statistical analysis was performed using Mann–Whitney (*n* = 3). **(G)** Chemotaxis index of human neutrophils toward LB, 0.02% PA in LB, 0.02% PAA in LB, and 0.02% PP in LB in *ex vivo* Transwell migration assays. LB broth was used to determine statistical significance (^∗^*P* < 0.05) with one-way ANOVA by Sidak’s comparison (*n* = 3). **(H)** Chemotaxis index of human neutrophils of A118 0.02% PA CFCM, 0.02% PAA CFCM, or 0.02% PP CFCM with or without PF induction. Statistical significance (^∗^*P* < 0.05) was performed using two-way ANOVA by Tukey’s comparison (*n* = 3), where bracketed bars with (^∗^) represent statistically significantly different index means. Single bars with (^∗^) represent statistically significant by comparison compared to controls. All *ex vivo* Transwell migration assay chemotaxis indexes were determined by dividing number of live neutrophils that migrated to the experimental condition (PA, PAA, PP, PA CFCM, PAA CFCM, and PP CFCM) divided by number of live neutrophils that migrated to LB broth.

qPCR of *paaA*, revealed a 2.28-fold decrease (*P* < 0.05) in transcription with or without PF (Figure 5A). In *A. baumannii*, Δ*paaA*, and Δ*paaE* deletion mutants were unable to grow in L-PA as sole carbon source (Cerqueira et al., 2014). Considering that the PAA catabolic pathway is a substantial shuttle for PA degradation, we expected PA to be an unfavorable growth substrate under PF induction. The 24-h growth curves of *A. baumannii* strain A118 in 0.2% PA minimal media revealed a slight reduction in growth between LB and PF conditions, and a minimal growth in 0.2% PAA for both conditions (Figure 5B). However, our phenotype microarray revealed that when L-PA was used as the sole carbon source, OD_590_ values of PF-induced cells were sustained (Figure 2). This can be attributed to the upregulation of three of the five aromatic amino acid transporters (Supplementary Table S1) that can take up PA.

Sustained growth in L-PA, despite the RNA-seq downregulation of peg.705, *feaB*, and the *paa* locus, suggests that *A. baumannii* utilizes an alternative pathway for PA catabolism. This was supported by the qPCR for *paaA* gene expression, as well as the sustained growth of A118 when LB was supplemented with 0.2% PA, but a lack of growth when supplemented with PAA (Figure 5C). Our data also suggest the presence of an alternative pathway in strains A42 and AB5075, which displayed similar patterns of growth when PA was used as sole carbon source (Supplementary Figures S3B,C).

A feasible alternative route for PA catabolism is its transamination to PP. We found two genes, peg.2520 and peg.3587, encoding proteins with PA transamination activity that were upregulated in the presence of PF. When PP was used as a sole carbon source, we observed a decrease in growth of PF-induced samples (Figure 5B), and when LB was supplemented with 0.2% PP, the growth pattern did not change (Figure 5C). The reduction in growth in PP + PF minimal media may be attributed to the energy barrier associated with favoring alternative pathways of PA degradation other than the PAA catabolic route [42], which involve oxidative decarboxylation of PP to phenylacetylaldehdye, phenylacetate, hydroxyl-3-phenylpropionate, and/or 2-hydroxyphenylacetate.

Among the four possible routes, only the gene encoding 4-hydroxyphenylpyruvate dioxygenase, the enzyme responsible for 2-hydroxyphenylacetate formation, was differentially expressed. The corresponding gene, *hpd*, peg.2560, was upregulated by 1.83-fold in PF (Supplementary Table S1). The qPCR of *hpd* revealed a 20.9-fold increase in LB + PF transcripts (Figure 5D). Considering our transcriptomic results, A118 in the absence of PF may not favor PP conversion to 2-hydroxyphenylacetate *via hpd*, and the increased growth pattern of PP (Figure 5B) can be attributed to a preferential shift toward the three other possible routes that may be more energetically efficient (Figure 5E).

2-Hydroxyphenylacetate is both a substrate in tyrosine metabolism and styrene degradation. A central part of styrene degradation is the production of homogentisate from 2-hydroxyphenylacetate and 3-hydroxyphenylacetate, followed by a conversion to 4-maleylacetoactate. The enzymes responsible for this process have not been described in *A. baumannii*; however, in our phenotype microarray, we found a 1.39-fold increase in growth for A118 PF in 3-hydroxyphenylacetic acid (Figure 2). We also observed a 1.19-fold increase in growth in L-tyrosine as an additional nitrogen source (Figure 2), suggesting that production and utilization of 2-hydroxyphenylacetate are favored in the presence of PF for A118.

The next step of styrene degradation is the isomerization of 4-maleyacetoacetate to fumarylacetoacetate *via* malelylacetoacetate isomerase. Fumarylacetoacetate is then cleaved by fumarylacetoacetase into fumarate and acetoacetate to be shuttled into the TCA cycle and propionate metabolism, respectively. The genes encoding both malelylacetoacetate isomerase (peg.2257), fumarylacetoacetase (peg.2556), and fumarate hydratase class I (*fumA*, peg.2808) were upregulated by PF, and propionate metabolism was shown to be favored under PF induction (Supplementary Table S1 and Figure 2). Moreover, growth of A118 PF was 1.32-fold higher when grown on acetoacetic acid (Figure 2). Altogether, these results suggest an alternative pathway for PA degradation through the transamination to PP with the derivatives of the pathway shuttling the TCA cycle and propionate metabolism, rather than the use of the PAA catabolic route (Figure 5E).

### PF’s Impact on PA Catabolism Triggers a Neutrophil-Evasive State in *A. baumannii*

Recently, the *A. baumannii* PAA degradation route of PA was reported to play a significant role in the mediation of neutrophil chemotaxis. PAA was found to be a powerful neutrophil chemoattractant in *ex vivo* experiments of mouse-derived neutrophils, and Δ*paaA* deletion mutants increased *in vivo* neutrophil migration and dwelling in zebrafish^15^. With these findings in mind, we hypothesized that under PF induction, the combined down- and upregulation of the PAA and PP degradation routes of PA, respectively, inhibits the accumulation and release of PAA in strain A118. Hence, this relatively avirulent strain would then be able to make use of the energetic advantages of host-derived PA degradation using the proposed alternative PA pathway while simultaneously reducing host neutrophil recruitment.

To evaluate the role of PA metabolism in neutrophil recruitment, we performed *ex vivo* neutrophil chemotaxis assays using human-derived primary neutrophils. Growth curves of PA and its derivatives revealed no significant changes in the growth pattern between PA + LB and PA + PF (Figure 5F), indicating that addition of the essential amino acid (PA) in the presence of PF may have an effect beyond growth augmentation given our RNA-seq data. All conditions reached stationary phase above 1.0 OD_600_ within 5 h; however, a 1.37-fold reduction in growth for A118 0.02% PP + PF was observed at stationary phase compared to all other conditions. This pattern is consistent with the reduction observed in minimal media testing (Figure 5B), indicating that the PP alternative pathway may not be the most energetically optimal route. Therefore, we hypothesize that the growth pattern during the lag phase represents the quick degradation of PP followed by a less energetically efficient process of shuttling alternative pathway intermediates to growth-promoting energy processes. Despite the limited efficiency, the PP alternative route may be the most favorable in terms of survival in the host as it limits PAA accumulation.

In the *ex vivo* chemotaxis assays, when LB was supplemented with PA, PAA, or PP, neutrophil migration increased by more than 2-fold compared to LB for both PA and PAA conditions. Interestingly, in the presence of PP, chemotaxis increased by 4-fold (Figure 5G). These results suggest that the alternative PA pathway induced by PF is a peripheral response to uptake and degrade PP to prevent neutrophil recruitment and may account for the significant upregulation of *hpd*.

When neutrophil chemotaxis assays were performed using A118 CFCM, PA + PF CFCM resulted in a significant 2.01-fold reduction in neutrophil migration compared to PA + LB CFCM (Figure 5H), confirming our hypothesis that PF triggers the incorporation of exogenous PA while simultaneously prompting a neutrophil evasive state. Therefore, the proposed alternative pathway for PA catabolism is most likely a selective process that is strengthened by the uptake and degradation of PP. While not as energetically efficient as the PAA pathway, this alternative strategy probably exerts the largest survival payoff—neutrophil evasion. This is especially highlighted in the 2.21-fold reduction in chemotaxis for PP + PF CFCM (Figure 5H). In fact, PP + LB CFCM chemotactic levels matched that of PP control, indicating that PP uptake and degradation by A118 are not favored in the absence of PF. This is further illuminated by the slightly higher chemotaxis observed in PF + PAA CFCM compared to PA + PF CFCM and PP + PF CFCM, which may account for the downregulation of the *paa* operon and subsequent PAA catabolic route. However, PF + PAA CFCM still demonstrated a reduction in chemotaxis by 1.46-fold compared to LB + PAA CFCM, which may be attributed to PAA’s relationship to tyrosine metabolism, to which we observed higher growth in the phenotype microarray (Figure 1A, 5E). These results represent a significant finding, as neutrophils are considered key actors in *A. baumannii* infection clearance [39]. The potential reduction of bacterial metabolite chemoattractant observed under the influence of a serous fluid not only highlights the pathoadaptability of *A. baumannii* but also establishes a link between metabolism and virulence/persistence features.

### The Expression of Additional Genes Involved in Central Metabolism Is Also Affected by PF in *A. baumannii* Strain A118

**FIGURE 6:**
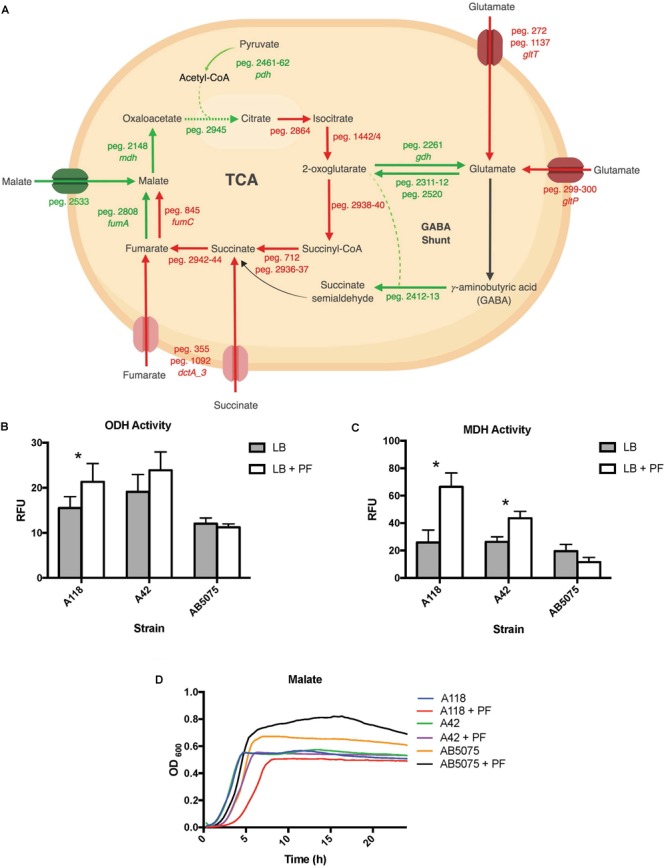
PF induces changes in expression of genes involved in metabolism. **(A)** Genes differentially expressed by PF linked to the TCA cycle and GABA shunt. Red words and arrows depict statistically significant downregulated genes (adjusted *P* < 0.05). Green words and arrows depict statistically significant upregulated genes (adjusted *P* < 0.05). Dotted arrows indicate DEGs that were not statistically significant. Black arrows indicate genes that were not differentially expressed under PF treatment. Dashed lines indicate a second substrate utilized in the enzymatic conversion to next metabolite of the pathway. **(B)** 2-oxoglutarate dehydrogenase (ODH) activity of the cell soluble extracts of three model strains, indicated by relative fluorescent units (RFU). RFU values were recorded with an excitation of 340 and emission of 460 to qualify NADH formation, indicating activity of the dehydrogenase. Statistically significant results (^∗^*P* < 0.05; multiple t-test; *n* = 3–6). **(C)** Minimal media (MM) growth curves of strains A118, A42, and AB5075 in 0.2% malate and 0.2% fumarate with or without PF. Mann–Whitney statistical analysis was performed to analyze all growth assays (*P <* 0.05; *n* = 3). **(D)** Malate dehydrogenase (MDH) activity of cell soluble extracts of the three model strains of *Acinetobacter baumannii*. Statistically significant results (^∗^*P* < 0.05; multiple *t*-test; *n* = 3–6).

Analyzing the transcriptomic data related with metabolic processes and focusing our analysis on genes involved in central metabolism, we observed that 13 of the 17 DEGs linked to the tricarboxylic acid (TCA) cycle were downregulated upon PF exposure (Supplementary Table S1 and Figure 6A). Interestingly, 11 of the 12 genes involved in the stepwise formation of fumarate from citrate were downregulated (Figure 1B), including aconitate hydratase I (peg.2864), isocitrate dehydrogenase (peg.1442/4), the 2-oxoglutarate dehydrogenase complex (peg.2938–40), succinyl-CoA synthetase (peg.712), succinyl-CoA ligase (peg.2936–37), and succinate dehydrogenase (peg.2941–44). Following the production of fumarate, all aerobic enzyme-associated genes of the TCA cycle were upregulated, including fumarate hydratase class I (peg.2808) and MDH (peg.2148). However, the fumarate hydratase class II (peg.845), the fumarase favored under oxidative and iron-limiting stress, and two of three genes encoding C4-dicarboxlayte transporters, peg.1092 and peg.355, were downregulated by PF (Supplementary Table S1).

These transcriptional changes were confirmed by our phenotype microarray data, which showed a reduction in bacterial growth rate when citric acid, succinic acid, and α–ketoglutaric acid (the conjugate base of 2-oxoglutarate) were used a sole carbon sources, and an increase in bacterial growth rate when racemic or L-malic acid were used as sole carbon sources for LB + PF-induced cells (Figure 2).

The 2-oxoglutarate dehydrogenase complex was selected for dehydrogenase activity testing due to 2-oxoglutarate’s link to glutamate metabolism, which we hypothesized to be a significant pathway in *A. baumannii*’s response to PF due to the profound growth observed on L-glutamic acid (Figure 2). A118 transcriptomic data revealed that 14 genes associated with glutamate metabolism were upregulated in the presence of PF (Supplementary Table S1). The upregulation of *gdh* (peg.2261) in particular may account for the higher NADH formation (1.4-fold increase) observed when soluble extracts of A118 incubated with PF were supplemented with 2-oxoglutarate (Figure 6B) despite the downregulation of the 2-oxogluatrate complex, for glutamate dehydrogenase is responsible for 2-oxoglutarate conversion to glutamate.

Glutamate metabolism plays an essential role in the microbial response to acid and oxidative stress, particularly through γ-aminobutyrate (GABA) catabolism (Feehily and Karatzas, 2013). GABA is a known component of the GABA shunt, which utilizes glutamate to produce succinate, bypassing certain steps of the TCA cycle. Remarkably, the gene encoding γ-aminobutyrate-α-ketoglutarate aminotransferase (GABA-T), peg.2412, was upregulated by 8.18-fold in response to PF. This upregulation was phenotypically confirmed by the higher OD_590_ observed when γ-aminobutyrate was used as the sole carbon source (Figure 2). As the more virulent strains of *A. baumannii* (A42 and AB5075) did not exhibit statistically significant increases in NADH formation in 2-oxoglutarate dehydrogenase assays in the presence of PF (Figure 6B), our results suggest a strain-specific secondary pathway for 2-oxoglutarate utilization favoring glutamate metabolism in less virulent *A. baumannii* strains. Further studies including more low virulent strains are needed to support the premise presented.

In addition, the *mdh* (MDH) genes (peg.2148) and a malic acid transporter encoding gene (peg.2533) were upregulated (Supplementary Table S1). MDH assays showed statistically significant increases in NADH production for both A118 (2.56-fold) and A42 (1.65-fold) strains in the presence of PF, while AB5075 demonstrated a slight reduction (Figure 6C). Interestingly, growth curves of PF-induced *A. baumannii* in L-malic acid demonstrated an extended lag phase in A118 that diminished in A42 and was absent in AB5075 (Figure 6D). We stress that among the three tested strains, A118 is the most susceptible and least virulent, A42 is mildly susceptible and virulent, and AB5075 is the most resistant, highly virulent strain (Table 1), and subsequently, as pathogenicity increased, PF-induced MDH activity significantly reduced and the lag phase in L-malic growth curves shortened. Taken together, these observations suggest that malate is a significant substrate in a strain-specific metabolic persistence strategy elicited by PF similar to that observed in PYR-induced persister formation.

## Discussion

### PF-Induced Metabolic Strategies and Survival

*Acinetobacter baumannii* is known to be an extremely adaptable pathogen due to its genome plasticity, as well as its metabolic versatility upon exposure to host effectors. In the presence of mucin, a glycoprotein secreted by lung epithelial cells, virulence features associated with the *paa* operon, as well benzoate metabolism, amino acid and lipid catabolism, electron transport activity, and peptide transport are upregulated (Ohneck et al., 2018). HSA, a component of PF, was previously reported to promote expression of arginine metabolism and succinyl-CoA production genes, to which the latter was linked to polypetide biosynthesis, a metabolite that can confer a competitive advantage during pathogenesis (Allen and Gulick, 2014; Quinn et al., 2018c).

In this work, we observed a profound transcriptional response elicited by PF, especially within genes involved in metabolism in *A. baumannii* A118, which suggests a metabolically driven strategy for survival during respiratory infection. This included opposing transcriptional responses in lipid metabolism genes characterized by downregulation of lipid biosynthesis and upregulation of short chain fatty acid (propionate) degradation. Based on the observed downregulation of genes associated with fatty acid synthesis, we propose that *A. baumannii* may obtain fatty acids from PF to exert negative feedback on ACC and the FASII system to reduce *de novo* lipid biosynthesis in *A. baumannii*.

In this study, PF induction also revealed a relationship between PYR, a critical factor in energy production and central sugar metabolism, to different modes of survival. We showed that the addition of PYR alters cytotoxic activity of secreted molecules against human epithelial cells (Figure 3A,B). In *S. aureus*, PYR causes global changes to the exoproteome that were then correlated to enhanced cytotoxicity against polymorphonuclear neutrophils (Harper et al., 2018). Sensor kinase two-component systems (SK-TCS) conserved in the *Staphylococcus* genus were implicated in this modulation of virulence; accordingly, we propose that host-derived PYR activates sensory systems that work in conjunction with the effects of PF on the global regulation of *A. baumannii*’s virulence.

By implicating PYR in the *A. baumannii* survival strategy, this study strikingly demonstrates an essential link to the pathogen’s adaptable physiology, as this metabolite is essential not only for host energy production but also for maintaining neutrophil immunonutrition (Mathioudakis et al., 2011). When testing the role of PYR in antibiotic persistence, we found that as the pathogenicity of the tested strain increases (antibiotic resistance and virulence), the positive rescue effects of PYR decrease in the presence of PF. Opposing metabolic strategies between resistant and susceptible strains of *A. baumannii* have been previously reported, specifically within TCA metabolite abundance levels using untargeted metabolomics. Maifiah et al. (2016) found that polymyxin-susceptible strains revealed higher abundance of early TCA cycle intermediates, while resistant strains demonstrated higher abundance of late TCA cycle intermediates (Maifiah et al., 2016). Our TCA cycle differential expression analysis revealed an upregulated transcriptomic response toward late cycle intermediates. We also observed a similar strain-specific strategy in MDH activity as the effect observed in the antibiotic persistence model upon the addition of PYR.

The reduced pathogenicity together with increased metabolic activity for PYR utilization provide an interesting link between differential metabolism, persistence, and virulence. The observed correlation between PF-mediated modulation of MDH activity and *A. baumannii* pathogenicity indicates the divergence in survival strategies between different strains, as the enzyme and its substrates are correlated to biofilm production (Shin et al., 2009) and are connected to the end products of the proposed alternative PA catabolism pathway for neutrophil immune evasion (Figure 5E).

### Immune Evasion Strategy Stimulated by PF

Neutrophils are extremely potent immune effectors against pathogens and are necessary to coordinate an effective response to various immune cells (Mccracken and Allen, 2014). The granulocytes are essential agents against *A. baumannii* infection, as the bactericidal mechanisms of nitric oxide synthase 2 and NADP oxidases have been shown to be critical in host protection from the pathogen (Qiu et al., 2009). In murine respiratory infection models, neutrophil depletion resulted in lethal *A. baumannii* infections with enhanced bacterial burdens of the lung that were associated with delayed production of pro-inflammatory cytokines such as tumor necrosis factor-α (TNF-α, interleukin-6 (IL-6), KCP, monocyte chemoattractant protein-1 (MCP-1), and macrophage inflammatory protein-2 (MIP-2) (Van Faassen et al., 2007; Tsuchiya et al., 2012). Therefore, *A. baumannii* evasion against these potent effectors would be extremely advantageous in the survival of the pathogen.

Bhuiyan et al. (2016) reported that the metabolic by-product of the *paa* catabolic pathway of PA has a significantly positive neutrophil chemotactic effect. Therefore, the proposed alternative PA degradation pathway induced by PF in A118 constitutes a feasible immune evasion strategy against neutrophils in *A. baumannii*.

The dramatic 4-fold increase in chemotaxis of neutrophils in PP + LB and subsequent reduction in the A118 CFCM induced by PF suggest that PP is a significant immunoregulatory metabolite in human neutrophils. Macrophage migration inhibitory factor (MIF) is a PP tautomerase cytokine that uses PP and p-hydroxyphenylpyruvate as substrates during the innate immune response (Rosengren et al., 1996, 1997). MIF has been hypothesized to play an essential role in innate immunity regulation, as its production has been shown to be induced by Gram-negative endotoxins, TNF-α and interferon-γ (INF-γ) (Calandra and Roger, 2003). The cytokine promotes the expression of a number of proinflammatory molecules including cytokines, NO, matrix metalloproteinases, COX-2, and other arachidonic acid pathway products (Mitchell et al., 1999, 2002). In fact, rat *in vivo* models have directly correlated MIF to the accumulation of activated neutrophils through regulation of MIP-2 (also known as CXCL-2), a neutrophil chemoattractant cytokine (Baugh and Bucala, 2002).

Given that macrophages constitute 75% of the white blood cell count of PF, we propose that the amplified PP degradation and reduced chemotaxis obtained under PF induction are direct responses by *A. baumannii* to reduce MIF’s substrate availability. Therefore, by upregulating tyrosine and PA alternative metabolism, the pathogen can prevent neutrophil activation and recruitment by reducing macrophage cytokine signaling.

Neutrophils are produced and stored in large reserves in the bone marrow, ready to be deployed into the circulation upon cytokine stimulation (Yamashiro et al., 2001). Therefore, tissue resident macrophages are the first innate immune effectors to respond to infection. These cells are less immunoreactive than neutrophils, making their primary purpose to signal for more potent immune effectors (Davies and Taylor, 2015). In fact, alveolar macrophages were shown to require 24 more hours to phagocytose and kill 80% of *A. baumannii*, while neutrophils were able to clear *A. baumannii* within 1 h (Qiu et al., 2009). However, the same study demonstrated enhanced susceptibility of mice to *A. baumannii* challenge during *in vivo* depletion of alveolar macrophages. This can be attributed not only to the bactericidal activity of macrophages but also to the activation of the secretion of neutrophil-recruiting chemokines such as CXCL-1, MIP-2, IL-1α, and MCP-1 (Beck-Schimmer et al., 2005; De Filippo et al., 2008; Barry et al., 2013). Strains resistant to early innate immune factors, like tissue-resident macrophages, have been shown in *A. baumannii* to enhance bacterial burden *in vivo* and have been correlated to the virulence state (Bruhn et al., 2015). Therefore, the enhanced cytotoxicity of PF-induced A118 CFCM against macrophages (Figure 3C,D) could be a survival strategy not only to prevent killing but also to prevent the recruitment of the more potent neutrophil. Accordingly, our observations provide evidence of novel mechanisms of pathoadaptability of *A. baumannii* using host signals to modify its virulence state.

The first line of infection is the epithelial cell, and cytoadherence, which is an important process in *A. baumannii* virulence (Mortensen and Skaar, 2012), has been pointed out as a significant stimulus for the production of proinflammatory cytokines in human lung epithelial cells (Yang et al., 2003). Cytoadherence, while enhancing the pathogenesis of *A. baumannii* infection, can be disadvantageous due to the apoptotic factor signaling of phagocytic effectors. By releasing cytotoxic exoproteins, *A. baumannii* would have the opportunity to promote its survival by preventing invasion-induced sacrificial apoptosis, as well as the recruitment of activated innate immune effectors. Therefore, the enhanced cytotoxicity effect observed against epithelial cells may be the first component of a neutrophil evasion strategy during *A. baumannii* respiratory infection.

**FIGURE 7:**
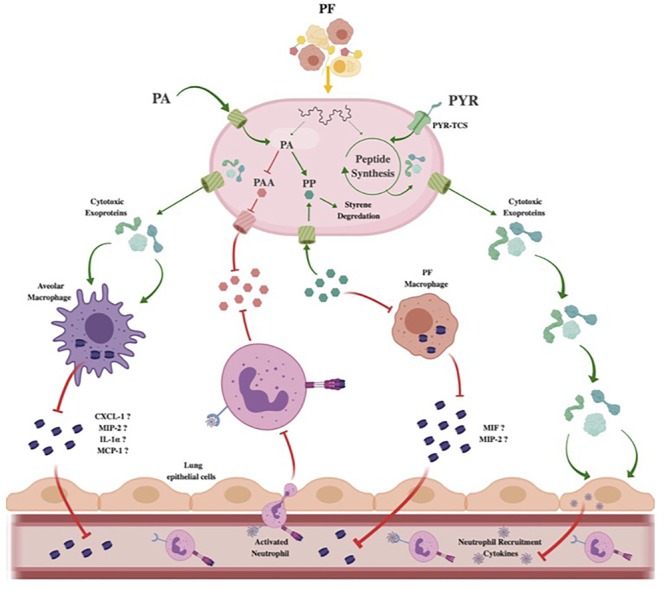
Theoretical model of *Acinetobacter baumannii* neutrophil evasion strategy elicited by PF. In the host respiratory space, *A. baumannii* encounters PF, inducing a survival-based transcriptional response. PA uptake and alternative catabolism toward styrene degradation are amplified to prevent PP accumulation. The halting of PP release inhibits PF-resident macrophages from utilizing the phenylpyruvate tautomerase cytokine macrophage inhibitory factor (MIF). Lack of active MIF reduces mobilization of MIP-2, a known neutrophil recruiting cytokine. Concurrently, the PAA catabolic route for PA degradation is downregulated to prevent bacterial metabolite-driven neutrophil chemoattraction. Resident respiratory space macrophages (pictured as alveolar macrophages) are then targeted by secreted cytotoxic exoproteins induced by PF, preventing pathogen recognition and subsequent secretion of proinflammatory chemokines such as CXCL-1, MIP-2, IL-1α, and MCP-1. Simultaneously, host-derived PYR is sensed by a membrane two-component system transport protein (PYR-TCS) to be used in conjunction with the effects of PF to produce epithelial cell-specific cytotoxic proteins. Epithelial cell (pictured as lung epithelial cells) inflammatory response is then reduced, inhibiting second-wave neutrophil recruitment. Red inhibitory lines represent downregulated/inhibited processes. Green arrows and proteins represent upregulated processes.

For both PYR and PA, we showed how acquiring these metabolites from the external environment aids *A. baumannii*’s survival and virulence and proposed that they are used under the induction of PF to promote a neutrophil immune evasion during respiratory infection (Figure 7). Interestingly, both PYR and PA have a metabolic pathway connected to propionate metabolism *via* acetyl-CoA interconversion to malonate semialdehyde and PP conversion to fumarylacetoacetate, respectively (Figure 1C, 5E). Propionate and many of the intermediates associated with its biosynthesis, including acetoacetic acid, malonic acid, and L-valine, were observed to have increased utilization in the presence of PF (Figure 2 and Supplementary Figure S4), and we observed an upregulation of propionate metabolism genes (Supplementary Table S1). Propionate has been shown to dampen the cytokine and nitric oxide response of mouse and human monocytes and macrophages to microbial stimulation (Ciarlo et al., 2016). Considering the short chain fatty acid’s connection to both PA and PYR metabolism under PF induction observed here, propionate may serve as a central link for further exploration into our proposed immune evasion strategy enacted by *A. baumannii*.

In the present work, we mimic aspects of bacterial–host interactions by exposure to PF and observed that *A. baumannii* cytotoxicity and immune evasion are enhanced in response to changes in intracellular PYR and PA metabolism. The differential expression of genes involved in these metabolic processes was also affected upon PF exposure, leading to enhanced cytotoxicity against human cells and weakened human neutrophil chemotaxis, as well as the augmented occurrence of persister cells. Together, these results indicate that metabolism, immune evasion, and persistence strategies exhibited by *A. baumannii* exist on a multidimensional axis linked by external environmental signals in response to a harsh host environment.

## Data Availability

The datasets generated for this study can be found in the Gene Expression Omnibus (GEO), GSE131949.

## Author Contributions

NR, JM, JF, SF, and MR conceived the study and designed the experiments. JM, JF, NR, CL, SM, ED, AMM, JN, ALM, CH, RS, and MR performed the experiments and genomics and bioinformatics analyses. NR, JM, JF, SF, VJ, RB, RS, and MR analyzed the data and interpreted the results. NN, CB, VJ, and MR contributed reagents, materials, and analysis tools. NR, JM, JF, VJ, CB, RB, RS, and MR wrote the manuscript. All authors read and approved the final version of the manuscript.

## Conflict of Interest Statement

The authors declare that the research was conducted in the absence of any commercial or financial relationships that could be construed as a potential conflict of interest.

## Footnotes

1 http://www.bioinformatics.babraham.ac.uk/projects/fastqc/

## Supplementary Material

FIGURE S1 Bioinformatic analysis of RNA-seq data from LB- or 4% PF-treated *Acinetobacter baumannii* A118. **(A)** PCA plot of all RNA-seq samples. Biological replicates of the same treatment are indicated by color in the legend. **(B)** Heat map of the expression profiles of the 500 most highly expressed genes based on DESeq log_2_ transformation of read count data. Gene- (rows) and sample-wise (columns) clustering dendrograms are shown.

FIGURE S2 Differential gene expression of *Acinetobacter baumannii* A118 in response to PF. Volcano plot showing log_2_fold changes vs. *P*-value for all genes of *A. baumannii* A118. Black dots depict genes with differences below the threshold limit of significance (adjusted *P* < 0.05), indicated by a blue dashed line. Genes within the area of statistically significant differential downregulation or upregulation higher than 2-fold change are depicted as red and green dots, respectively. Genes within the area of statistically significant differential downregulation or upregulation between 1.5- and 2-fold change are depicted as light red and light green dots, respectively. Dots corresponding to genes involved in all metabolic pathways described in this analysis are outlined in blue. Dots corresponding to specific pathways of interest are highlighted in the indicated colors.

FIGURE S3 Growth curves under PF induction. **(A)** A118 grown in LB broth supplemented with 1% PYR with or without PF. **(B)** A42 grown in minimal media supplemented with 0.2% PA and 0.2% PAA with or without PF. **(C)** AB5075 grown in minimal media supplemented with 0.2% PA and 0.2% PAA with or without PF. Mann–Whitney statistical analysis was performed analyzing all growth assays (*P* < 0.05; *n* = 3).

FIGURE S4 Heat map of phenotype microarray. **(A,B)** A118 growth with or without PF induction on sole carbon sources and **(C)** sole nitrogen sources.

TABLE S1 Genes associated with metabolic processes with differential expression in *Acinetobacter baumannii* strain A118 under PF induction.
