# Supplementary figures and images for: Human Pleural Fluid Elicits Pyruvate and Phenylalanine Metabolism in Acinetobacter baumannii to Enhance Cytotoxicity and Immune Evasion
Source: Front Microbiol. 2019 Jul 17;10:1581. doi: 10.3389/fmicb.2019.01581 (PMC6650585; doi:10.3389/fmicb.2019.01581)

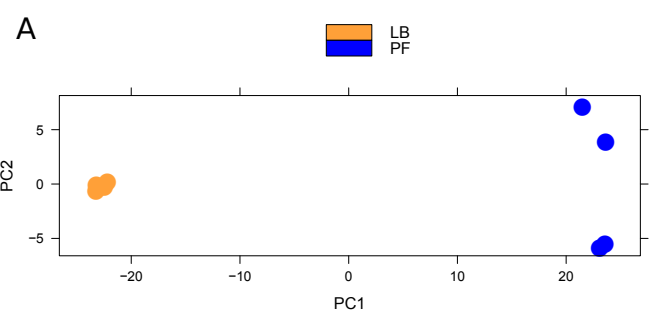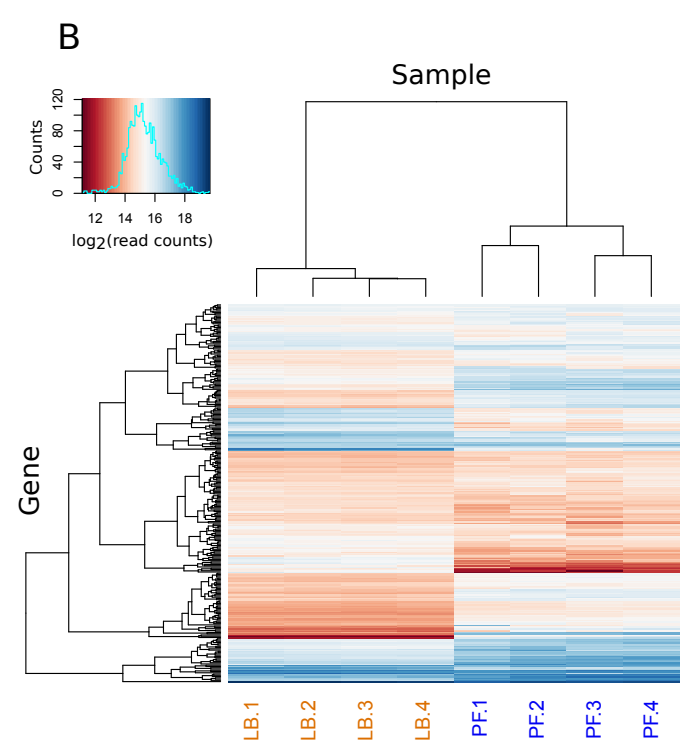

Supplement: FIGURE S1 — Bioinformatic analysis of RNA-seq data from LB- or 4% PF-treated Acinetobacter baumannii A118. (A) PCA plot of all RNA-seq samples. Biological replicates of the same treatment are indicated by color in the legend. (B) Heat map of the expression profiles of the 500 most highly expressed genes based on DESeq log2 transformation of read count data. Gene- (rows) and sample-wise (columns) clustering dendrograms are shown. [file Image_1.pdf]

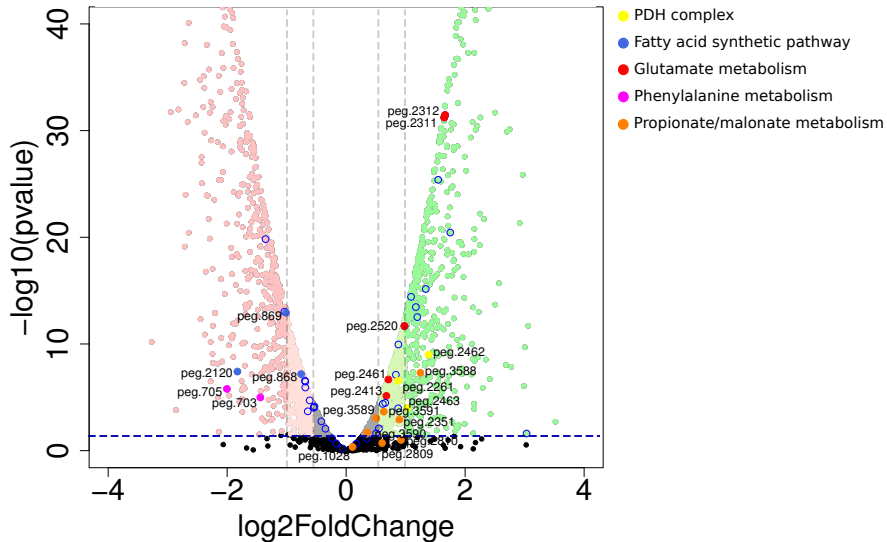

Supplement: FIGURE S2 — Differential gene expression of Acinetobacter baumannii A118 in response to PF. Volcano plot showing log2fold changes vs. P-value for all genes of A. baumannii A118. Black dots depict genes with differences below the threshold limit of significance (adjusted P < 0.05), indicated by a blue dashed line. Genes within the area of statistically significant differential downregulation or upregulation higher than 2-fold change are depicted as red and green dots, respectively. Genes within the area of statistically significant differential downregulation or upregulation between 1.5- and 2-fold change are depicted as light red and light green dots, respectively. Dots corresponding to genes involved in all metabolic pathways described in this analysis are outlined in blue. Dots corresponding to specific pathways of interest are highlighted in the indicated colors. [file Image_2.pdf]

**(A)**

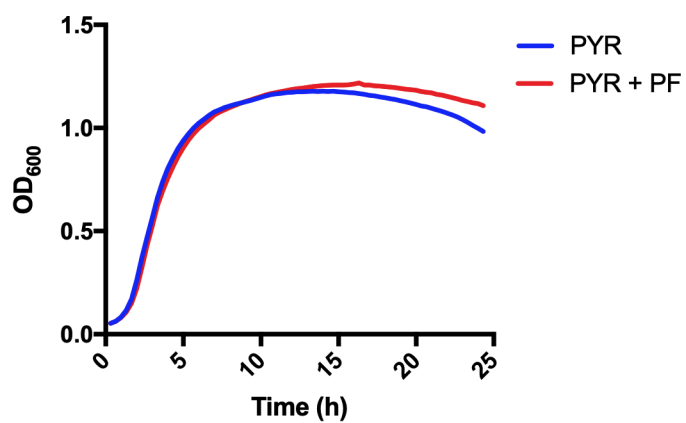

**(B)**

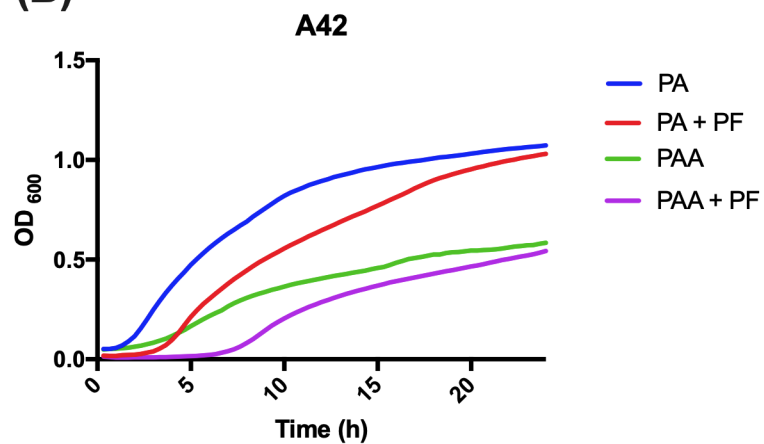

**(C)**

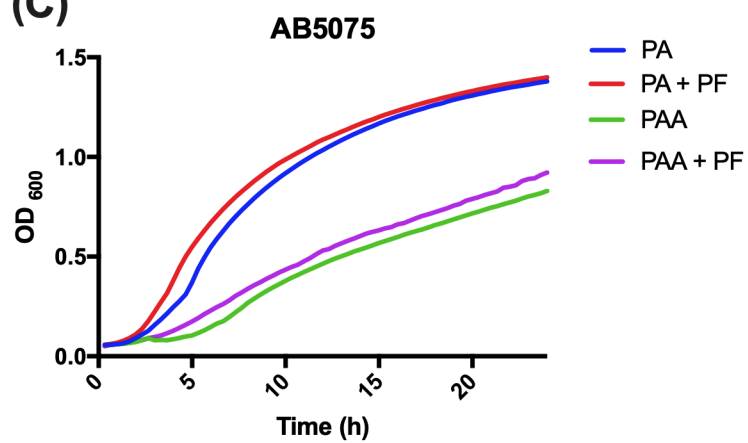

Supplement: FIGURE S3 — Growth curves under PF induction. (A) A118 grown in LB broth supplemented with 1% PYR with or without PF. (B) A42 grown in minimal media supplemented with 0.2% PA and 0.2% PAA with or without PF. (C) AB5075 grown in minimal media supplemented with 0.2% PA and 0.2% PAA with or without PF. Mann–Whitney statistical analysis was performed analyzing all growth assays (P < 0.05; n = 3). [file Image_3.pdf]

(A)

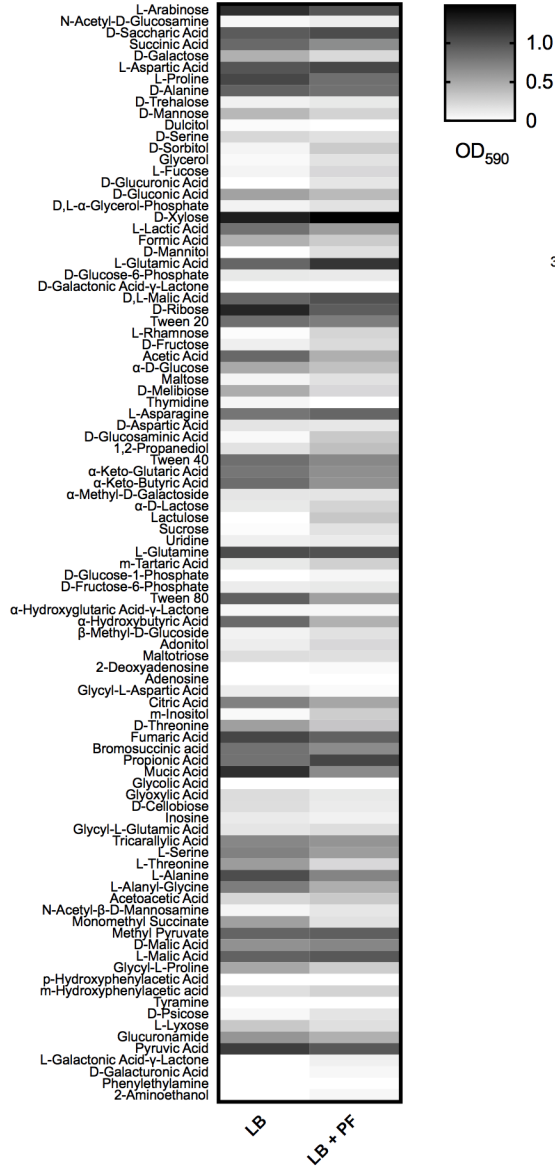

(B)

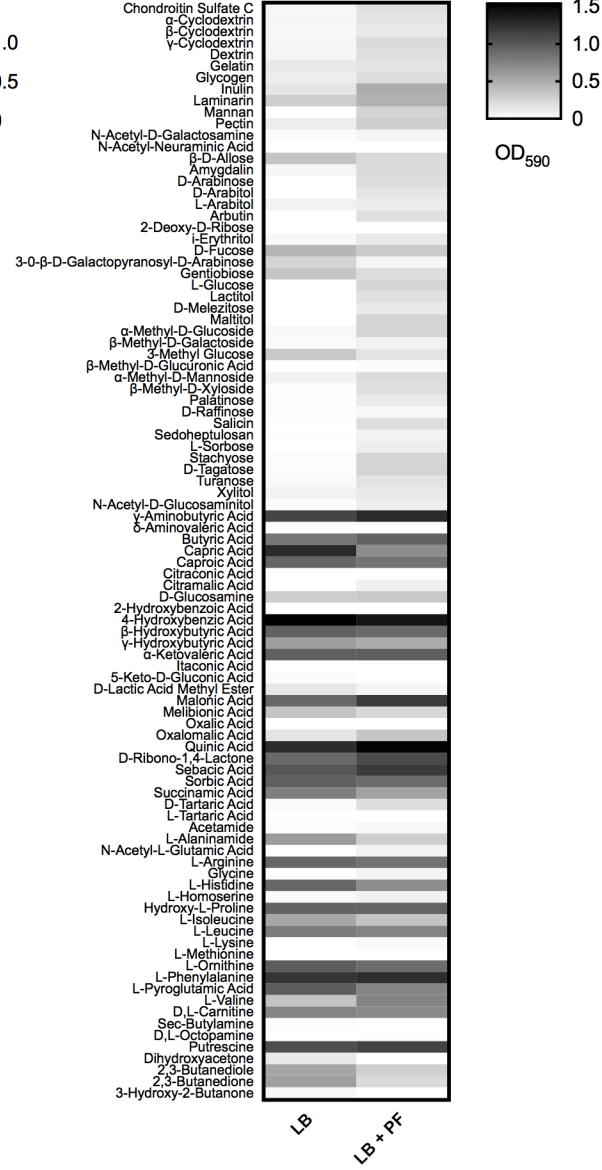

(C)

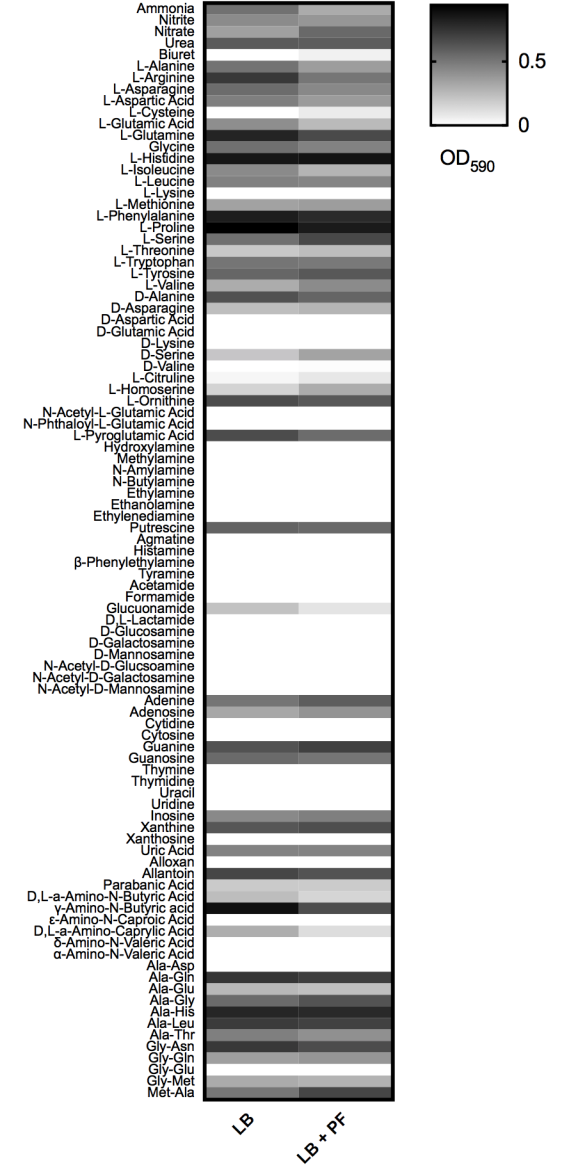

Supplement: FIGURE S4 — Heat map of phenotype microarray. (A,B) A118 growth with or without PF induction on sole carbon sources and (C) sole nitrogen sources. [file Image_4.pdf]
