# Supplementary material for: Human Pleural Fluid Elicits Pyruvate and Phenylalanine Metabolism in Acinetobacter baumannii to Enhance Cytotoxicity and Immune Evasion
Source: Front Microbiol. 2019 Jul 17;10:1581. doi: 10.3389/fmicb.2019.01581 (PMC6650585; doi:10.3389/fmicb.2019.01581)
Supplement: TABLE S1 — Genes associated with metabolic processes with differential expression in Acinetobacter baumannii strain A118 under PF induction. [file Table_1.DOCX]

| **Table 1:** |  |  |  |  |  |  | |
| --- | --- | --- | --- | --- | --- | --- | --- |
| **Gene Identifier** | **Gene Name** | **Fold Change** | **log_2_fold Change** | **P-value** | **Predicted Function** |  | |
| ***Tricarboxylic acid cycle (TCA) associated genes*** | | | | | |  |  |
| peg.2945 |  | 1.273498352 | 0.348797093 | 0.104050579 | Citrate synthase |  | |
| peg.2864 |  | 0.618956167 | -0.692090851 | 8.01E-07 | Aconitate hydratase I |  | |
| peg1442 |  | 0.639387678 | -0.645237154 | 4.33E-04 | Isocitrate dehydrogenase |  | |
| peg.1444 |  | 0.833872179 | -0.26210184 | 0.072863215 | Isocitrate dehydrogenase (monomeric) |  | |
| peg.2938 |  | 0.785827186 | -0.347716017 | 0.014622048 | Dihydrolipoamide dehydrogenase of 2-oxo-glutarate dehydrogenase complex | | |
| peg2939 |  | 0.749511499 | -0.415977482 | 0.003338576 | Dihydrolipoamide succinyl transferase E2 of 2-oxo-glutarate dehydrogenase complex |  | |
| peg.2940 |  | 0.691198299 | -0.532828427 | 0.000156324 | 2-oxoglutarate dehydrogenase E1 subunit |  | |
| peg.712 |  | 0.163512622 | -2.612526087 | 6.33E-68 | Succinyl-coA synthetase α-subunit |  | |
| peg.2936 | *sucD* | 0.621512699 | -0.686144226 | 9.76E-07 | Succinyl-coA ligase [ADP-forming] alpha chain |  | |
| peg.2937 | *sucC* | 0.590893046 | -0.759031075 | 6.93E-08 | Succinyl-coA ligase [ADP-forming] beta chain |  | |
| peg.2941 |  | 0.961297217 | -0.056945538 | 0.694544334 | Succinate dehydrogenase iron-sulfur protein |  | |
| peg.2942 | *sdhA* | 0.849544047 | -0.235239344 | 0.096810028 | Succinate dehydrogenase flavoprotein subunit |  | |
| peg.2943 | *sdhB* | 0.892057447 | -0.164791474 | 0.273135914 | Succinate dehydrogenase iron-sulfur subunit |  | |
| peg.2944 | *sdhC* | 1.126672589 | 0.172068329 | 0.279768063 | Succinate dehydrogenase cytochrome b-556 subunit |  | |
| peg.2808 | *fumA* | 2.538723907 | 1.344103506 | 4.68E-17 | Fumarate hydratase class I |  | |
| peg.845 | *fumC* | 0.597111409 | -0.743927961 | 1.46E-07 | Fumarate hydratase class II |  | |
| peg.2148 | *mdh* | 1.784693487 | 0.83567632 | 2.38E-07 | Malate dehydrogenase |  | |
| ***Glutamate metabolism genes*** | | | | | |  |  |
| peg.2312 |  | 3.169873348 | 1.664425199 | 7.40E-31 | Glutamate synthase [NADPH] large chain (EC 1.4.1.13) |  | |
| peg.2311 |  | 3.135027434 | 1.648478068 | 1.19E-30 | Glutamate synthase [NADPH] small chain (EC 1.4.1.13 |  | |
| peg.938 |  | 0.300684887 | -1.73367574 | 4.79E-30 | Ferredoxin-dependent glutamate synthase (EC 1.4.7.1) |  | |
| peg.3541 |  | 2.177158757 | 1.122446612 | 2.98E-14 | N-acetylglutamate synthase (EC 2.3.1.1) |  | |
| peg.2326 |  | 2.034996906 | 1.025026601 | 9.57E-13 | UDP-N-acetylmuramoylalanyl-D-glutamate-2,6-diamino-pimelate ligase (EC 6.3.2.13) |  | |
| peg.3499 |  | 2.621565596 | 1.390428645 | 1.04E-11 | UDP-N-acetylmuramoylalanine--D-glutamate ligase (EC 6.3.2.9) |  | |
| peg.2543 |  | 2.003176722 | 1.002289703 | 1.88E-11 | Glutamate racemase (EC 5.1.1.3) |  | |
| peg.3098 |  | 1.852696586 | 0.889626632 | 8.39E-10 | Dihydrofolate synthase (EC 6.3.2.12) / Folylpolyglutamate synthase (EC 6.3.2.17 |  | |
| peg.3136 |  | 1.790552533 | 0.840404846 | 1.12E-08 | Glutamate-ammonia-ligase adenylyltransferase (EC 2.7.7.42) |  | |
| peg.2261 |  | 1.636351651 | 0.710482816 | 6.31E-07 | NAD-specific glutamate dehydrogenase (EC 1.4.1.2) |  | |
| peg.1605 |  | 0.617918777 | -0.694510881 | 1.43E-06 | N-formylglutamate deformylase (EC 3.5.1.68) |  | |
| peg.2256 |  | 1.776665165 | 0.829171813 | 5.66E-05 | Succinylglutamate desuccinylase (EC 3.5.1.96) |  | |
| peg.3193 |  | 1.444915429 | 0.530985054 | 1.92E-04 | Glutamate N-acetyltransferase (EC 2.3.1.35) / N-acetylglutamate synthase (EC 2.3.1.1) |  | |
| peg.223 |  | 0.627319089 | -0.672728631 | 5.34E-04 | N-acetylglutamate synthase (EC 2.3.1.1) |  | |
| peg.2705 |  | 1.398789044 | 0.484178402 | 0.001732955 | Glutamate racemase (EC 5.1.1.3) |  | |
| peg.1429 |  | 0.794081254 | -0.332641458 | 0.026529978 | Glutamate-1-semialdehyde aminotransferase (EC 5.4.3.8) |  | |
| peg.2412 | *gabT* | 8.1850274 | 3.032987246 | 0.038449158 | Gamma-aminobutyrate:alpha-ketoglutarate aminotransferase (EC 2.6.1.19} |  | |
| peg.2413 | *gabR* | 1.598007339 | 0.676274034 | 1.83E-05 | Transcriptional regulator GabR of GABA utilization |  | |
| peg.2520 |  | 1.973695489 | 0.980899422 | 9.70E-12 | Aspartate aminotransferase (EC 2.6.1.1) |  | |
| ***Pyruvate Metabolism*** | | | | | |  |  |
| peg.1414 |  | 0.357978137 | -1.482056617 | 7.32E-18 | Pyruvate decarboxylase |  | |
| peg.2461 |  | 1.830003003 | 0.871846016 | 8.01E-07 | hypothetical E3 subunit of pyruvate dehydrogenase complex |  | |
| peg.2462 | *aceF* | 2.613686432 | 1.386086069 | 3.73E-09 | Dihydrolipoamide acetyltransferase E2 subunit of pyruvate dehydrogenase complex |  | |
| peg.2463 | *aceE* | 2.037545606 | 1.026832352 | 2.17E-04 | E1 subunit of pyruvate dehydrogenase subunit |  | |
| peg.3583 |  | 1.533255861 | 0.616598465 | 9.38E-05 | L-lactate permease |  | |
| peg.3584 |  | 2.52901013 | 1.338572816 | 4.44E-15 | Lactate-response regulator |  | |
| peg.3585 |  | 2.291685648 | 1.196409162 | 1.53E-12 | L-Lactate dehydrogenase |  | |
| peg.3586 |  | 2.253394198 | 1.172099714 | 1.93E-13 | D-Lactate dehydrogenase |  | |
| peg.997 |  | 0.68127711 | -0.553686359 | 2.37E-03 | Alcohol dehydrogenase |  | |
| peg.868 | *accB* | 0.592748878 | -0.754507068 | 2.13E-07 | Biotin carboxyl carrier protein of acetyl-CoA carboxylase |  | |
| peg.869 | *accC* | 0.496393615 | -1.010443538 | 6.10E-13 | Biotin carboxylase of acetyl-coA carboxylase |  | |
| peg.2120 | *accC* | 0.281972139 | -1.826375477 | 1.23E-07 | Biotin carboxylase of acetyl-CoA carboxylase (EC 6.3.4.14 |  | |
| peg.3099 | *accD* | 1.837600174 | 0.87782289 | 4.50E-10 | Acetyl-coenzyme A carboxyl transferase beta chain (EC 6.4.1.2) |  | |
| peg.2916 | *accA* | 1.01751051 | 0.025043695 | 0.894993901 | Acetyl-coenzyme A carboxyl transferase alpha chain (EC 6.4.1.2) |  | |
| peg.739 |  | 0.262985533 | -1.926944659 | 2.94E-33 | Acetyl-CoA acetyltransferase of β-ketoadipyl-CoA thiolase |  | |
| peg.701 |  | 0.227294918 | -2.137362665 | 5.82E-25 | Acetyl-CoA acetyltransferase of β-ketoadipyl-CoA thiolase |  | |
| ***Phosphoenolpyruvate: Carbohydrate Phosphotransferase System*** | | | | | |  |  |
| peg.2595 | *ppc* | 3.364039975 | 1.750194849 | 3.45E-20 | Phosphoenolpyruvate carboxylase |  | |
| peg.1063 | *ppsA* | 0.835069967 | -0.260031014 | 0.079509762 | Phosphoenolpyruvate synthase |  | |
| peg.1611 |  | 1.305299498 | 0.384380867 | 0.022063937 | Phosphoenolypyruvate carboxykinase GTP |  | |
| peg.797 |  | 0.887809512 | -0.171617 | 0.280794811 | Phosphoenolpyruvate-protein phosphotransferase of PTS system |  | |
| ***Propionate Metabolism*** | | | | | |  |  |
| peg.3588 |  | 2.375879906 | 1.248461914 | 1.60E-07 | Propionate catabolism operon transcriptional regulator of GntR family = transcriptional activator |  | |
| peg.3589 | *prpB* | 1.417236384 | 0.503080408 | 1.73E-03 | Methylisocitrate lyase |  | |
| peg.3590 | *prpC* | 1.27733981 | 0.353142375 | 0.031403466 | 2-methycitrate synthase |  | |
| peg.3591 | *prpD* | 1.550753321 | 0.632969215 | 4.63E-04 | 2-methylcitrate dehydratase |  | |
| peg.1028 | *acnB* | 1.0780076 | 0.108367349 | 0.521122081 | Aconitate hydratase II |  | |
| peg.2351 |  | 1.860241646 | 0.89549004 | 2.23E-03 | Propionyl-CoA:succinyl-CoA transferase |  | |
| peg.2810 |  | 1.898039862 | 0.924510292 | 0.137656935 | Acetate kinase |  | |
| peg.2809 |  | 1.519314149 | 0.603420207 | 0.251788689 | Phosphate acetyltransferase |  | |
| ***Malonate Metabolism*** | | | | | |  |  |
| peg.1150 |  | 1.399682741 | 0.485099855 | 0.001559579 | Methylmalonate-semialdehyde dehydrogenase |  | |
| peg.236 |  | 1.136934385 | 0.185148996 | 0.602721508 | Malonate utilization transcriptional regulator |  | |
| peg.2881 |  | 1.536614061 | 0.61975486 | 2.26E-05 | Enoyl-CoA hydratase |  | |
| peg.3117 |  | 1.715538029 | 0.778661107 | 2.98E-08 | Acyl-CoA dehydrogenase (EC 1.3.8.7) |  | |
| ***Aminotransferase genes*** | | | | | |  |  |
| peg.2412 | *gabT* | 8.1850274 | 3.032987246 | 0.038449158 | Gamma-aminobutyrate:alpha-ketoglutarate aminotransferase (EC 2.6.1.19) |  | |
| peg.2413 |  | 1.598007339 | 0.676274034 | 1.83E-05 | Transcriptional regulator GabR of GABA utilization (GntR family with aminotransferase-like domain) |  | |
| peg.1151 |  | 1.57690872 | 0.657099151 | 7.52E-05 | Omega-amino acid--pyruvate aminotransferase (EC 2.6.1.18) |  | |
| peg.3135 |  | 1.356424185 | 0.439808412 | 0.002295863 | Branched-chain amino acid aminotransferase (EC 2.6.1.42) |  | |
| peg.3587 |  | 2.922862986 | 1.547382202 | 5.41E-25 | Biosynthetic Aromatic amino acid aminotransferase alpha (EC 2.6.1.57) |  | |
| peg.2542 |  | 2.323481421 | 1.216288109 | 5.17E-18 | Glucosamine--fructose-6-phosphate aminotransferase (EC 2.6.1.16) |  | |
| peg.3286 |  | 2.198759084 | 1.136689538 | 1.69E-12 | Adenosylmethionine-8-amino-7-oxononanoate aminotransferase (EC 2.6.1.62) |  | |
| peg.2520 |  | 1.973695489 | 0.980899422 | 9.70E-12 | Aspartate aminotransferase (EC 2.6.1.1) |  | |
| peg.2581 |  | 1.540257501 | 0.623171562 | 3.99E-05 | Transcriptional regulator GntR family domain / Aspartate aminotransferase (EC 2.6.1.1) |  | |
| peg.3471 |  | 1.500538107 | 0.585479958 | 0.001165097 | Transcriptional regulator GntR family domain / Aspartate aminotransferase (EC 2.6.1.1) |  | |
| peg.1473 |  | 1.257580684 | 0.330650964 | 0.021965667 | Aspartate aminotransferase (EC 2.6.1.1) |  | |
| ***Phenylacetate Catabolic Route*** | | | | | |  |  |
| peg.705 |  | 0.249502053 | -2.002876409 | 4.38E-06 | Monoamine oxidase (EC 1.4.3.4) |  | |
| peg.703 | *feaB* | 0.368119443 | -1.441754144 | 2.43E-05 | Phenylacetaldehyde dehydrogenase (EC 1.2.1.39) |  | |
| peg.143 | *paaK* | 1.004444452 | 0.006397782 | 0.921630566 | Phenylacetate-coA ligase |  | |
| peg.134 | *paaA* | 0.686222746 | -0.543251148 | 0.003837678 | Phenylacetyl-coA epoxidase subunit A |  | |
| peg.135 | *paaB* | 0.651974754 | -0.617111993 | 0.000118746 | Phenylacetyl-coA epoxidase subunit B |  | |
| peg.136 | *paaC* | 0.607661263 | -0.718660769 | 2.97E-07 | Phenylacetyl-coA epoxidase subunit C |  | |
| peg.137 | *paaD* | 0.791450275 | -0.337429384 | 0.03502945 | Phenylacetyl-coA epoxidase subunit D |  | |
| peg.138 | *paaE* | 0.775777041 | -0.366286015 | 0.077990078 | Phenylacetyl-coA expoxidase subunit E |  | |
| peg.140 | *paaG* | 0.734287336 | -0.445583377 | 0.001447178 | ring 1,2 epoxyphenylacetyl-coA isomerase PaaG |  | |
| peg.133 | *paaZ* | 0.712531346 | -0.488974612 | 0.006411923 | Phenylacetic acid degradation, aldehyde dehydrogenase PaaZ |  | |
| peg.142 | *paaJ* | 0.716969103 | -0.480017145 | 0.000508068 | Phenylacetic acid degradation protein PaaJ |  | |
| peg.146 | *paaI* | 1.606802274 | 0.684192408 | 0.017617348 | Acyl-coenzyme A thioesterase PaaI |  | |
| peg.145 | *paaY* | 0.895557246 | -0.159142439 | 0.375890267 | Phenylacetic acid degradation protein PaaY |  | |
| peg.139 | *paaF* | 0.829760966 | -0.269232303 | 0.101271101 | 2,3-dehydroadipyl-coA hydratase PaaF |  | |
| peg.141 | *paaH* | 0.776801441 | -0.364382218 | 0.008687542 | 3-hydroxyladipyl-coA dehydrogenase PaaH |  | |
| peg.144 | *paaX* | 1.05225087 | 0.073478703 | 0.828029613 | Transcriptional repressor of *paa* catabolic operon PaaX |  | |
| peg.712 |  | 0.163512622 | -2.612526087 | 6.33E-68 | Succinyl-CoA synthetase alpha subunit |  | |
| peg.2936 | *sucD* | 0.621512699 | -0.686144226 | 9.76E-07 | Succinyl-coA ligase [ADP-forming] alpha chain |  | |
| peg.2937 | *sucC* | 0.590893046 | -0.759031075 | 6.93E-08 | Succinyl-coA ligase [ADP-forming] beta chain |  | |
| ***Phenylpyruvate Catabolic Route*** | | | | | |  |  |
| peg.2520 | *aspC* | 1.973695489 | 0.980899422 | 9.70E-12 | α-keto transaminase |  | |
| peg.3587 | *tyrB* | 2.922862986 | 1.547382202 | 5.41E-25 | Biosynthetic Aromatic amino acid aminotransferase alpha (EC 2.6.1.57) |  | |
| peg.2560 | *hpd* | 1.833372142 | 0.874499657 | 2.35E-04 | 4-hydroxyphenylpyruvate dioxygenase (EC 1.13.11.27) |  | |
| peg.2557 | *hmgC* | 1.464927209 | 0.55082898 | 0.01 | Maleylacetoacetate isomerase (EC 5.2.1.2) |  | |
| peg.2556 | *hmgB* | 1.418211268 | 0.504072464 | 0.02 | Fumarylacetoacetase (EC 3.7.1.2) |  | |
| peg.2808 | *fumA* | 2.538723907 | 1.344103051 | 4.68E-17 | Fumarate hydratase class I%2C aerobic (EC 4.2.1.2) |  | |
| peg.2148 | *mdh* | 1.784693487 | 0.83567632 | 2.38E-07 | Malate dehydrogenase (EC 1.1.1.37) |  | |
| ***Aromatic Amino Acid Transporters*** | | | | | |  |  |
| peg.1413 |  | 0.527565502 | -0.922577867 | 3.87E-06 | Aromatic amino acid transport protein AroP |  | |
| peg.2414 |  | 4.397761523 | 2.136769373 | 0.03 | Aromatic amino acid transport protein AroP (possible pheP) |  | |
| peg.2555 |  | 1.462358982 | 0.54829751 | 0.06 | Aromatic amino acid transport protein AroP |  | |
| peg.1894 |  | 1.193518977 | 0.255221506 | 0.16 | Aromatic amino acid transport protein AroP |  | |
| peg.2997 |  | 0.833878402 | -0.262091072 | 0.17 | Aromatic amino acid transport protein AroP |  | |
| ***Type II Fatty Acid Synthesis*** | | | | | |  | |
| peg.868 | *accB* | 0.592748878 | -0.754507068 | 2.13E-07 | Biotin carboxyl carrier protein of acetyl-CoA carboxylase |  | |
| peg.869 | *accC* | 0.496393615 | -1.010443538 | 6.10E-13 | Biotin carboxylase of acetyl-coA carboxylase |  | |
| peg.2120 | *accC* | 0.281972139 | -1.826375477 | 1.23E-07 | Biotin carboxylase of acetyl-CoA carboxylase (EC 6.3.4.14 |  | |
| peg.3099 | *accD* | 1.837600174 | 0.87782289 | 4.50E-10 | Acetyl-coenzyme A carboxyl transferase beta chain (EC 6.4.1.2) |  | |
| peg.2916 | *accA* | 1.01751051 | 0.025043695 | 0.894993901 | Acetyl-coenzyme A carboxyl transferase alpha chain (EC 6.4.1.2) |  | |
| peg.3297 | *fabD* | 1.424107508 | 0.510058062 | 3.81E-04 | FabD: Malonyl CoA-acyl carrier protein transacylase |  | |
| peg.233 | *fabD* | 0.700637834 | -0.5132592 | 7.23E-03 | FabD: Malonyl CoA-acyl carrier protein transacylase |  | |
| peg.1981 | *fabH* | 1.262559652 | 0.33635155 | 0.021657157 | FabH: 3-oxoacyl-[acyl-carrier-protein] synthase 3 (EC 2.3.1.41) |  | |
| peg.3335 | *fabB* | 0.898205063 | -0.15488321 | 0.321616271 | FabH: 3-oxoacyl-[acyl-carrier-protein] synthase 1 (EC 2.3.1.41) |  | |
| peg.192 | *fabG* | 0.201684787 | -2.30982583 | 2.01E-21 | FabG: 3-oxoacyl-[acyl-carrier-protein] reductase (EC 1.1.1.100) |  | |
| peg.709 | *fabG* | 0.186339518 | -2.423994426 | 1.14E-24 | FabG: 3-oxoacyl-[acyl-carrier-protein] reductase (EC 1.1.1.100) |  | |
| peg.953 | *fabG* | 0.626092353 | -0.675552614 | 2.33E-06 | FabG: 3-oxoacyl-[acyl-carrier-protein] reductase (EC 1.1.1.100) |  | |
| peg.1735 | *fabG* | 0.806410108 | -0.310414372 | 0.190733963 | FabG: 3-oxoacyl-[acyl-carrier-protein] reductase (EC 1.1.1.100) |  | |
| peg.1749 | *fabG* | 0.891953475 | -0.164959634 | 0.601671762 | FabG: 3-oxoacyl-[acyl-carrier-protein] reductase (EC 1.1.1.100) |  | |
| peg.2346 | *fabG* | 1.088097393 | 0.121807695 | 0.442249176 | FabG: 3-oxoacyl-[acyl-carrier-protein] reductase (EC 1.1.1.100) |  | |
| peg.2494 | *fabG* | 5.234860614 | 2.388151124 | 1.53E-40 | FabG: 3-oxoacyl-[acyl-carrier-protein] reductase (EC 1.1.1.100) |  | |
| peg.2887 | *fabG* | 0.785185318 | -0.348894899 | 0.038449158 | FabG: 3-oxoacyl-[acyl-carrier-protein] reductase (EC 1.1.1.100) |  | |
| peg.2975 | *fabG* | 1.170966732 | 0.227700088 | 0.152734019 | FabG: 3-oxoacyl-[acyl-carrier-protein] reductase (EC 1.1.1.100) |  | |
| peg.3298 | *fabG* | 0.983078842 | -0.02462097 | 0.871365619 | FabG: 3-oxoacyl-[acyl-carrier-protein] reductase (EC 1.1.1.100) |  | |
| peg.822 | *fabZ* | 1.188879689 | 0.249602726 | 0.092189879 | 3-hydroxyacyl-[acyl-carrier-protein] dehydratase |  | |
| peg.1458 | *fabK* | 0.383245224 | -1.38366028 | 1.36E-21 | Enoyl-[acyl-carrier-protein] reductase [FMN] (EC 1.3.1.9) |  | |
| peg.2840 | *fabI* | 1.101843031 | 0.139918711 | 0.352486103 | Enoyl-[acyl-carrier-protein] reductase [NADH] (EC 1.3.1.9) |  | |
| ***Exogenous Fatty Acid Utilization for Phospholipid Synthesis*** | | | | | |  | |
| peg.178 | *fadD* | 0.650157406 | -0.621139052 | 0.012840255 | Acetoacetyl-CoA synthetase (EC 6.2.1.16) / Long-chain-fatty-acid--CoA ligase |  | |
| peg.2724 | *fadD* | 1.043042759 | 0.060798302 | 0.786905752 | Acetoacetyl-CoA synthetase (EC 6.2.1.16) / Long-chain-fatty-acid--CoA ligase |  | |
| peg.3396 | *fadD* | 0.331762862 | -1.591775695 | 3.93E-21 | Long-chain-fatty-acid--CoA ligase (EC 6.2.1.3) |  | |
| peg.3463 | *fadD* | 2.240695723 | 1.16394675 | 1.18E-16 | Long-chain-fatty-acid--CoA ligase (EC 6.2.1.3) |  | |
| peg.2215 | *plsB* | 1.695139966 | 0.7614044 | 8.37E-08 | Glycerol-3-phosphate acyltransferase (EC 2.3.1.15) |  | |
| peg.58 | *plsC* | 1.520833576 | 0.604862289 | 3.57E-05 | 1-acyl-sn-glycerol-3-phosphate acyltransferase (EC 2.3.1.51) |  | |
| peg.1032 | *aas* | 0.50302189 | -0.99130691 | 4.33E-11 | 2-acylglycerophosphoethanolamine acyltransferase |  | |
|  |  |  |  |  |  |  | |
|  |  |  |  |  |  |  | |
|  |  |  |  |  |  |  | |
|  |  |  |  |  |  |  | |
|  |  |  |  |  |  |  | |
|  |  |  |  |  |  |  | |
|  |  |  |  |  |  |  | |
|  |  |  |  |  |  |  | |
|  |  |  |  |  |  |  | |
|  | | | | | |  |  |
|  |  |  |  |  |  |  | |
|  |  |  |  |  |  |  | |
|  |  |  |  |  |  |  | |
|  |  |  |  |  |  |  | |
|  |  |  |  |  |  |  | |
|  |  |  |  |  |  |  | |
|  |  |  |  |  |  |  | |
|  |  |  |  |  |  |  | |
